# A Cathepsin B‐Triggered CO‐Releasing Molecule with a Non‐Toxic Metal Core for Targeted Tumor Delivery

**DOI:** 10.1002/anie.202513808

**Published:** 2025-11-07

**Authors:** Inga Černauskienė, Eduardo Izquierdo‐García, Sarah Keller, Harley Betts, Kevin Cariou, Vicente Marchán, Gilles Gasser, Gonçalo J. L. Bernardes

**Affiliations:** ^1^ Yusuf Hamied Department of Chemistry University of Cambridge Lensfield Road Cambridge CB2 1EW UK; ^2^ Chimie ParisTech PSL University CNRS Institute of Chemistry for Life and Health Sciences Laboratory for Inorganic Chemical Biology Paris F‐75005 France; ^3^ Departament de Química Inorgànica i Orgànica Secció de Química Orgànica Universitat de Barcelona (UB) Institut de Biomedicina de la Universitat de Barcelona (IBUB) Martí i Franquès 1–11 Barcelona E‐08028 Spain; ^4^ Translational Chemical Biology Group Spanish National Cancer Research Centre (CNIO) C/ Melchor Fernández Almagro Madrid 3. 28029 Spain

**Keywords:** Bioconjugation, Bioorganometallic chemistry, Carbon monoxide, Cathepsin B, Tumor‐targeted therapy

## Abstract

Carbon monoxide (CO) has shown therapeutic potential across various diseases, including cancer. To enable controlled delivery, many CO‐releasing molecules (CORMs) have been developed. However, their clinical translation has been limited due to concerns about stability, potential toxicity, and insufficient targeting ability. In this study, we report the synthesis and characterization of an enzyme‐triggered CO‐releasing molecule (**ET‐CORM**) that can be site‐specifically conjugated to antibodies. This novel **ET‐CORM** is built on a biocompatible iron core, and releases CO upon cleavage by the cancer‐associated protease cathepsin B (CatB). The incorporation of a bioorthogonal handle into **ET‐CORM** enabled its efficient and site‐specific conjugation to the clinically used antibody trastuzumab via the interchain disulfide bonds. The resulting ET‐CORM–antibody conjugate (**ET‐CORM‐Ab**) exhibited an average drug‐to‐antibody ratio (DAR) of 6.8, corresponding to approximately 20 CO molecules per conjugate. This construct allowed for selective intracellular CO delivery to HER2‐overexpressing and CatB‐expressing cells in vitro. This study represents a metal‐based CORM–antibody conjugate activated by a tumor‐associated enzymatic trigger, opening new avenues for investigating CO‐mediated effects and advancing CO‐based cancer therapies to the clinics.

## Graphical abstract

An iron‐based, enzyme‐triggered CO‐releasing molecule (ET‐CORM) is site‐specifically conjugated to the antibody trastuzumab. This novel ET‐CORM‐antibody conjugate releases carbon monoxide upon cleavage by the tumor‐associated protease cathepsin B. The construct enables selective intracellular CO delivery to HER2‐overexpressing and CatB‐expressing cells, presenting a new strategy for targeted, CO‐based cancer therapies.

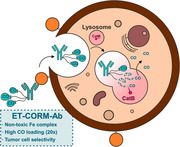

Carbon monoxide (CO) is endogenously produced through the breakdown of heme by heme oxygenase enzymes and functions as a potent gasotransmitter, alongside nitric oxide (NO) and hydrogen sulfide (H_2_S).^[^ ^1^ ^]^ Since this discovery, both physiological and pathological roles of CO have been reported.^[^ ^2^ ^]^ The therapeutic potential of CO has been demonstrated in a variety of contexts, including bacterial infections,^[^ ^3^, ^4^ ^]^ inflammatory diseases,^[^ ^5^, ^6^, ^7^ ^]^ organ transplantation,^[^ ^8^ ^]^ and cancer.^[^ ^9^, ^10^, ^11^, ^12^, ^13^, ^14^ ^]^ This broad therapeutic promise, along with the demonstrated safety of low‐dose inhaled CO, has driven the development of carbon monoxide‐releasing molecules (CORMs).^[^ ^15^, ^16^ ^]^ The earliest CORMs were metal‐ or boron‐based, using metals such as Cr, Co, Mo, Mn, Ru, and Re, with CO release triggered by external stimuli such as light, heat, nucleophiles, solvent, or pH changes.^[^ ^17^, ^18^, ^19^, ^20^, ^21^, ^22^, ^23^, ^24^, ^25^, ^26^ ^]^ Targeted CO delivery to tumors has been achieved using metal‐based CORMs complexed with non‐specific histidine residues of bovine serum albumin (BSA), enhancing tumor biodistribution and increasing half‐life,^[^ ^27^, ^28^ ^]^ or antibody‐photoCORM conjugates connected by streptavidin‐biotin system.^[^ ^29^ ^]^ Concerns over metal‐associated toxicity^[^ ^30^ ^]^ have led to the development of organic CO prodrugs, which generally offer lower CO payload by weight percentage, or sometimes have undefined by‐products.^[^ ^31^, ^32^, ^33^, ^34^, ^35^ ^]^ A major conceptual advance was introduced by Schmalz and co‐workers, who pioneered iron‐based enzyme‐triggered CORMs (ET‐CORMs). These systems enable controlled intracellular CO delivery in response to specific enzymatic activities, with notable examples activated by esterases, phosphatases, penicillin G amidase, and plasmin.^[^ ^36^, ^37^, ^38^ ^]^ Because iron is physiologically abundant, these complexes exhibit markedly lower toxicity than CORMs based on other metals. Subsequent studies by Schmalz and colleagues further demonstrated that ET‐CORMs possess good plasma stability and produce therapeutic effects consistent with the established biological benefits of CO.^[^ ^39^, ^40^, ^41^, ^42^, ^43^, ^44^, ^45^ ^]^

Building on the promising therapeutic potential of iron‐based enzyme‐triggered CORMs, we hypothesized that their targeting capabilities could be further enhanced through rational molecular design. Specifically, we proposed that incorporating a cathepsin B (CatB) cleavage site would enable tumor‐specific intracellular CO release, while introducing a bioorthogonal handle would allow tumor targeting through conjugation to a suitable antibody (Scheme 1). In this study, we describe the design and synthesis of a bioconjugatable, CatB‐activated CORM (**ET‐CORM**), along with its antibody conjugate, **ET‐CORM–Ab**, prepared using the HER2‐targeting antibody trastuzumab. **ET‐CORM–Ab** enabled selective intracellular CO delivery to HER2‐overexpressing SKBR3 cells in vitro. This represents the first example of a metal‐based CORM–antibody conjugate activated by a cancer‐related protease and directed against a cancer‐specific receptor, highlighting its potential for investigating the therapeutic effects of site‐specific CO delivery.

**Scheme 1:**
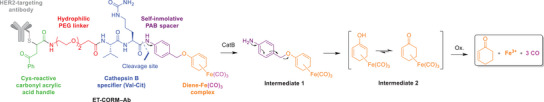
Structure of the HER2‐targeted **ET‐CORM–Ab** conjugate, highlighting key structural components and illustrating the cathepsin B–triggered 1,6‐elimination mechanism of the PAB spacer leading to CO release. This scheme is adapted from Refs. [37, 38], where the CO‐release mechanism was originally proposed.

To develop our bioconjugatable, protease‐triggered CORM, we designed a modular construct comprising a well‐characterized diene–Fe(CO)_3_ core^[^ ^36^, ^37^, ^38^ ^]^ as the CO donor, linked to the CatB–cleavable Val‐Cit dipeptide via a self‐immolative *para*‐aminobenzyl (PAB) spacer, using an approach previously validated by Schmalz and co‐workers through their development of plasmin‐triggered CORMs.^[^ ^37^, ^38^ ^]^ The Val‐Cit dipeptide motif was selected for its widespread use in several FDA‐approved ADCs (Adcetris, Padcev, and Polivy), owing to its excellent plasma stability and efficient intracellular cleavage by CatB, a lysosomal cysteine protease overexpressed in several invasive and metastatic cancers.^[^ ^46^, ^47^, ^48^, ^49^ ^]^ To improve aqueous solubility, a short polyethylene glycol chain was appended to the *N*‐terminus of the dipeptide, which was further functionalized with a carbonyl acrylic acid (CAA) handle^[^ ^50^, ^51^ ^]^ to allow irreversible site‐selective antibody conjugation by a Michael addition reaction with cysteine residues.

Using a synthetic strategy analogous to that reported in Ref. [38], commercially available Alloc‐Val‐Cit‐PAB‐OH was first converted into the corresponding benzyl chloride **4** by treatment with thionyl chloride in THF (Scheme 2). Subsequent coupling of **4** with the cyclohexadiene–Fe(CO)_3_ complex **5** (prepared according to Ref. [36]) in THF afforded intermediate **6** in 44% yield. Removal of the Alloc protecting group from complex **6** using Pd(PPh_3_)_4_ and diethylamine as a scavenger, yielded the corresponding free amine **7**. Finally, the target **ET‐CORM** was obtained in 37% yield via HATU‐mediated amide coupling of amine **7** with carboxylic acid **3**. Precursor **3** was synthesized by coupling activated ester **2** (prepared as described in Ref. [52]) with compound **1**, obtained via acid‐mediated hydrolysis of the corresponding *tert*‐butyl ester. All compounds were purified by silica gel column chromatography or reversed‐phase HPLC, fully characterized by high‐resolution ESI mass spectrometry, as well as by ^1^H and ^13^C NMR spectroscopy, and their purity was confirmed by analytical HPLC.

**Scheme 2:**
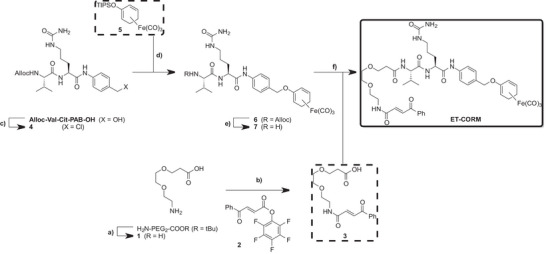
Synthetic strategy for the preparation of **ET‐CORM**. Reagents and conditions: a) TFA/CH_2_Cl_2_ (1:1), rt, 4 h, quant.; b) perfluorophenyl (*E*)‐4‐oxo‐4‐phenylbut‐2‐enoate (compound **2** ^[^ ^52^ ^]^), NEt_3_, CH_2_Cl_2_, rt, overnight, 78%; c) SOCl_2_, THF, rt, 4 h, quant.; d) compound **5**,^[^ ^36^ ^]^ TBAF, NaH, THF, 0  °C, 10 min; then compound **4**, THF, 0 °C to rt, overnight, 44%; e) [Pd(PPh_3_)_4_], diethylamine, THF, rt, overnight, 68%; f) compound **3**, HATU, DIPEA, DMF, rt, 4 h, 37%.

Having established an efficient synthetic route to access **ET‐CORM,** we next investigated whether CO release could be triggered by CatB‐mediated cleavage (Scheme 1). To prevent unwanted side reactions of the CAA moiety of **ET‐CORM** with nucleophiles present in the medium during the enzymatic assay, we prepared the **ET‐CORM–NAC** model compound by reacting ET‐CORM with *N*‐acetylcysteine (NAC) (Figure S1). This construct was then incubated with CatB (1 µg mL^−1^) using a previously reported assay (MES buffer, pH 5.5, 10 mM DTT).^[^ ^53^ ^]^ After 1 hr, only trace amounts of **ET‐CORM–NAC** and **Intermediate 1** remained, with complete disappearance observed after 2 h (Figures S2 and S3). Concurrently, **Intermediate 2** was detected, as indicated by a shift in retention time on UPLC‐MS and a corresponding mass signal observed exclusively in negative ESI mode. Upon addition of an excess oxidant to quench the high DTT concentration present in the buffer, **Intermediate 2** also disappeared (Figures S2 and S3). Lastly, CO release from **ET‐CORM–NAC** was confirmed using fluorescent CO‐sensitive probe **1‐Ac**,^[^ ^54^ ^]^ with the rate of CO release shown to be dependent on CatB concentration (Figure S4). The cleavage of the Val‐Cit‐PAB linker and CO release from the iron core are consistent with previously reported kinetics.^[^ ^37^, ^48^ ^]^

Next, we evaluated the cytotoxicity and CO‐releasing capability of **ET‐CORM** in two CatB‐expressing breast cancer cell lines, SKBR3 and MCF7, which are reported to have comparable CatB expression levels.^[^ ^55^, ^56^ ^]^ In both models, **ET‐CORM** exhibited low cytotoxicity over 48 h (IC_50_ > 100 µM, Figure 1a), similar to previously reported enzyme‐triggered CORMs bearing the same iron core. The CO release of these compounds after self‐immolation is reported to be slow, on the order of hours,^[^ ^37^ ^]^ resulting in low cytotoxicity. Such slow release is advantageous in contexts where CO acts synergistically, for example as an anti‐inflammatory agent with immunostimulatory potential.^[^ ^12^, ^13^, ^57^, ^58^, ^59^ ^]^ Intracellular CO release triggered by endogenous CatB was confirmed by a significant increase in turn‐on fluorescence of the CO‐sensitive probe **1‐Ac** ^[^ ^54^ ^]^ in both cell lines (Figures 1b,c, S5, and S9a). As expected, the **1‐Ac** signal in CatB‐low HEK293T cells was much lower (Figure S6), confirming that CO release depends on CatB concentration.

**Figure 1:**
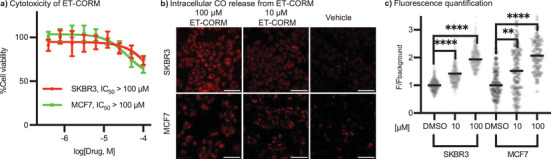
a) Cytotoxicity studies of **ET‐CORM** confirmed an IC_50_ > 100 µM in 48‐hour assay, consistent with previously reported iron‐based CORMs (24‐hour assay).^[^ ^45^ ^]^ b) Confocal microscopy images showing cellular CO release triggered by endogenous cathepsin B in SKBR3 and MCF7 cells treated with **ET‐CORM** (100 µM or 10 µM) or vehicle control (DMSO, dimethyl sulfoxide). Cells were pre‐treated with 5 µM of the CO‐sensitive probe **1‐Ac** for 30 min, followed by **ET‐CORM** or vehicle treatment for 60 min. Cells were then fixed and imaged. An increase in fluorescence indicates the turn‐on response of the **1‐Ac** CO probe (*λ*_ex_ = 561 nm, *λ*_em_ = 570–620 nm). Scale bar: 100 µm. c) Quantification of fluorescence from panel **b** and additional biological replicates. Background normalization refers to the median of the vehicle control. Significant turn‐on fluorescence of the **1‐Ac** CO probe was observed in both SKBR3 and MCF7 cells treated with 100 µM and 10 µM **ET‐CORM**, compared to the vehicle control. Statistical significance was determined using an unpaired t‐test and is indicated as **** (*p* < 0.00005), ** (*p* < 0.005), and ns (not significant; *p* > 0.05).

The role of CO in oncology is particularly complex,^[^ ^2^, ^60^ ^]^ underscoring the need for careful selection of targeting systems and for defining therapeutically relevant CO concentrations for each cancer type. Currently, targeting human epidermal growth factor receptor 2 (HER2) is a benchmark strategy in the development of antibody–drug conjugates (ADCs), especially when evaluating new conjugatable drugs or linker technologies. HER2 is overexpressed in certain types of cancer, and the availability of both HER2‐high and HER2‐low cell lines, together with the clinical success of high drug‐to‐antibody ratio (DAR) trastuzumab conjugates, such as Enhertu (DAR 7.7), which also utilizes CatB–mediated drug release, makes HER2 an ideal model system for evaluating targeted CO delivery.^[^ ^35^, ^61^ ^]^ Achieving detectable intracellular CO concentrations requires both high DAR loading and high HER2 receptor expression. These factors are critical for generating CO levels within the detection range of sensitive CO probes, such as **1‐Ac**, which has a detection limit of 50 nM.^[^ ^54^ ^]^ Previous studies have shown that HER2 receptors in the HER2‐high SKBR3 cell line can be saturated at low nanomolar concentrations,^[^ ^62^ ^]^ supporting the feasibility of using this system for targeted CO release studies in cellular models.

In the next step, the CAA–bearing **ET‐CORM** payload was successfully conjugated to trastuzumab, yielding **ET‐CORM–Ab** with a conjugation yield of 68% (Figure 2a). The resulting conjugate achieved an average DAR of 6.8, corresponding to 20 CO molecules per antibody, as approximated by both mass spectrometry and quantification of SDS‐PAGE (Figure 2b–d). Stability studies confirmed that the conjugate remained intact for at least 48 h under physiological conditions (PBS, pH 7.4). No spontaneous, solvent‐triggered CO release was observed (Figure S7). Notably, the bioconjugate retained its binding affinity for HER2, as confirmed by flow cytometry using a fluorescent secondary antibody and trastuzumab as a control (Figure 2e–g). The trastuzumab control (red line) indicated that HER2 receptor expression in SKBR3 cells was over 100‐fold higher than in MCF7 cells, while the bioconjugate **ET‐CORM–Ab** exhibited a nearly equivalent binding profile to trastuzumab (gray shading).

**Figure 2:**
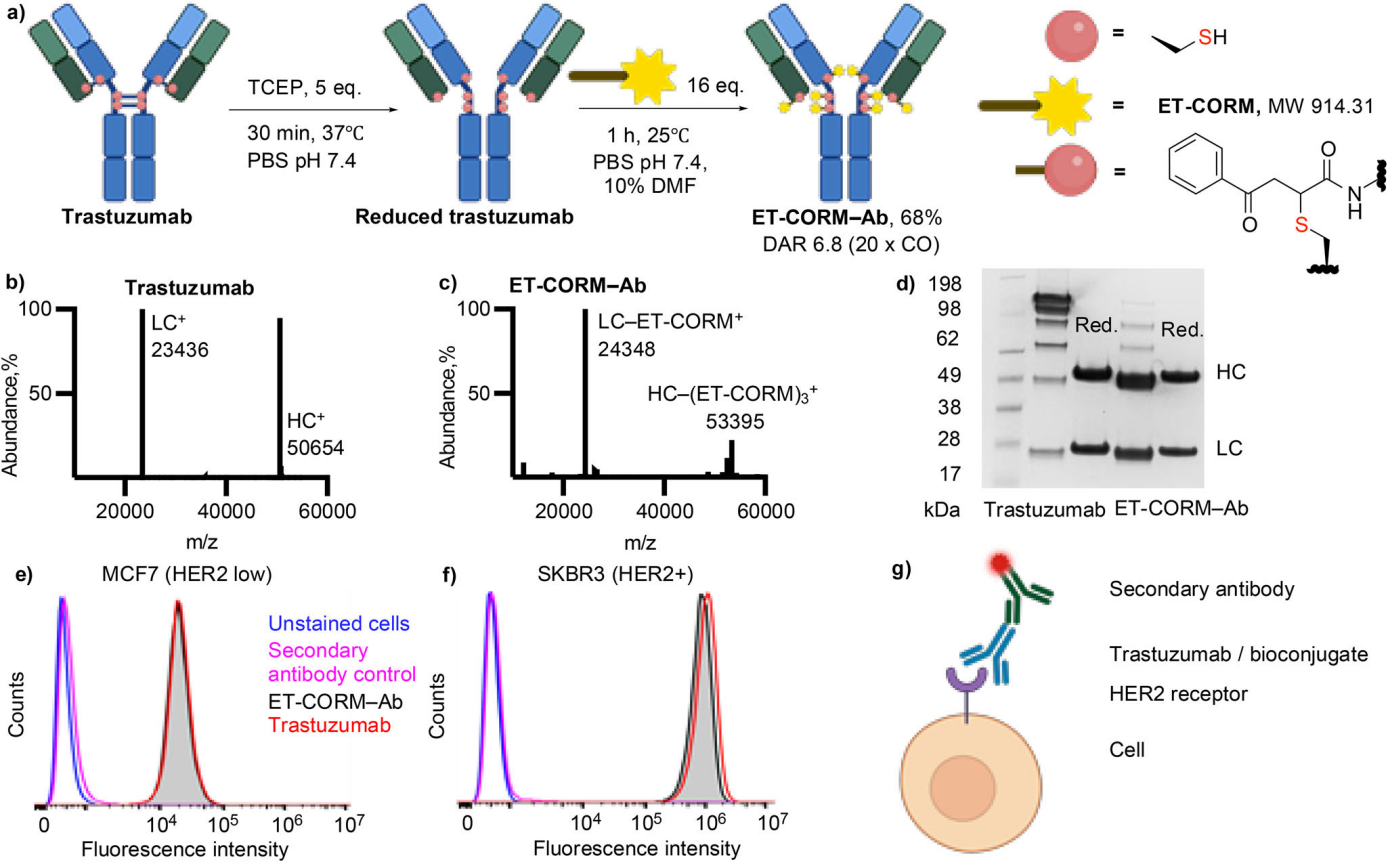
a) Schematic representation of trastuzumab bioconjugation via disulfide linkages. b) Deconvoluted reducing SQD mass spectrum of unmodified trastuzumab. c) Deconvoluted reducing SQD mass spectrum of **ET‐CORM–Ab** (calcd. LC 24,359 Da; HC (3 × modifications) 53,396 Da). d) SDS‐PAGE analysis of trastuzumab and **ET‐CORM–Ab** under both non‐reducing and reducing conditions (excess dithiothreitol, DTT). e)–g) Flow cytometry analysis of **ET‐CORM–Ab** binding specificity compared to trastuzumab in HER‐low MCF7 e), HER2‐high SKBR3 cells f) and schematic representation of the flow cytometry experiment g). Gray shading indicates **ET‐CORM–Ab** binding; blue line, unstained control; pink, secondary antibody control; red, trastuzumab binding (positive control). HC – heavy chain; LC – light chain; TCEP – tris(2‐carboxyethyl)phosphine; PBS – phosphate‐buffered saline; DMF – dimethylformamide. Cartoon figures created with *BioRender*.

With **ET‐CORM–Ab** in hand, we next sought to demonstrate selective CO release in HER2‐expressing cells using SKBR3 and MCF7 as representative models of high and low HER2 expression, respectively. CO release was detected using the fluorogenic CO sensor **1‐Ac**.^[^ ^54^ ^]^ A significant increase in fluorescence (*p* < 0.0005) was observed in **ET‐CORM–Ab**–treated SKBR3 cells at both 100 and 10 nM concentrations. In contrast, no significant fluorescence signals were detected under negative control conditions, including HER2 receptor blockade prior to **ET‐CORM–Ab** treatment and in the HER2‐low MCF7 cell line (Figure 3, Figures S8 and S9). Furthermore, similar to the non‐conjugated form, **ET‐CORM–Ab** showed no toxicity in SKBR3 cells (Figure S10), suggesting that the amount and rate of CO release may be insufficient to induce cell death.

**Figure 3:**
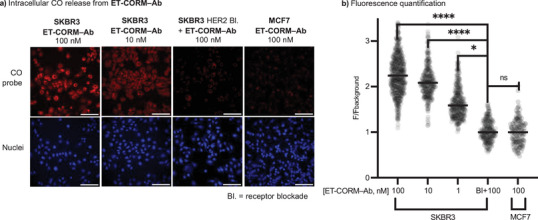
a) Confocal microscopy images showing intracellular CO release in SKBR3 (HER2‐high) and MCF7 (HER2‐low) cells treated with **ET‐CORM–Ab**. Cells were pre‐treated with 5 µM of the CO‐sensitive probe **1‐Ac** for 30 min, followed by incubation with **ET‐CORM–Ab** or control treatment for 2 h. After fixation, nuclei were stained with DAPI (blue), and intracellular CO release was visualized via the turn‐on fluorescence of the **1‐Ac** CO probe (red; *λ*_ex_ = 561 nm, *λ*_ex_ = 570–620 nm). Scale bar: 100 µm (white). b) Quantification of fluorescence from images shown in panel. a) A significant increase in **1‐Ac** probe fluorescence was observed in HER2‐high SKBR3 cells treated with **ET‐CORM–Ab** at concentrations ranging from 1 to 100 nM, while no significant fluorescence was detected in HER2‐low MCF7 cells. Pre‐treatment of SKBR3 cells with non‐fluorescent trastuzumab (HER2 receptor blockade) abolished the fluorescence response following 100 nM **ET‐CORM–Ab** treatment. Background normalization was performed relative to the median fluorescence of the vehicle control. Statistical significance was determined using an unpaired t‐test: **** (*p* < 0.00005), * (*p* < 0.05), and ns (not significant; *p* > 0.05).

In conclusion, we report the design, synthesis, characterization and functional validation of **ET‐CORM–Ab**, a CatB–activated, antibody‐conjugated carbon monoxide‐releasing molecule that enables targeted CO delivery to HER2‐overexpressing cancer cells. By combining the enzyme responsiveness of **ET‐CORM** with the tumor‐targeting specificity of trastuzumab, our platform achieves dual‐layer cancer selectivity, leveraging both HER2 overexpression and intracellular CatB activity for precise CO release. Using iron as the central metal ensures biocompatibility, while the Val‐Cit dipeptide enables enzyme‐specific intracellular activation. The high drug‐to‐antibody ratio (DAR 6.8) demonstrates substantial payload capacity, supporting the feasibility of achieving therapeutically relevant CO levels in a localized, controlled manner. We anticipate that this modular system can be advantageous in combination with long‐circulating antibody–drug conjugates, or immune checkpoint inhibitors, and it will facilitate mechanistic studies of low‐dose CO in cancer biology and inform future development of targeted gasotransmitter therapeutics.

## Supporting Information

The authors have cited additional references within the Supporting Information.^[^ ^63^ ^]^

## Conflict of Interests

The authors declare no conflict of interest.

## Supporting information



Supporting Information

## Data Availability

The data that support the findings of this study are available in the Supporting Information of this article.
