# Supplementary material for: A Cathepsin B‐Triggered CO‐Releasing Molecule with a Non‐Toxic Metal Core for Targeted Tumor Delivery
Source: Angew Chem Int Ed Engl. 2025 Nov 7;65(1):e13808. doi: 10.1002/anie.202513808 (PMC12759243; doi:10.1002/anie.202513808)
Supplement: Supplementary file 1 — Supporting Information [file ANIE-65-e13808-s001.pdf]

A Cathepsin B-Triggered CO-Releasing Molecule with a Non-Toxic Metal Core for Targeted Tumor Delivery

Inga Černauskienė,†[a] Eduardo Izquierdo-García,†[b,c] Sarah Keller,[b] Harley Betts,[b] Kevin Cariou,[b] Vicente Marchán[c], Gilles Gasser,*[b] Gonçalo J. L. Bernardes*[a, d]

[a] I. Cernauskiene, Prof. G. J. L. Bernardes
Yusuf Hamied Department of Chemistry, University of Cambridge, Lensfield Road, Cambridge, CB2 1EW, UK.
E-mail: gb453@cam.ac.uk

[b] Dr. E. Izquierdo-García, Dr. S. Keller, Dr. H. Betts, Dr. Kevin Cariou, Prof. G. Gasser
Chimie ParisTech, PSL University, CNRS, Institute of Chemistry for Life and Health Sciences, Laboratory for Inorganic Chemical Biology, F-75005 Paris, France. E-mail: gilles.gasser@chimieparistech.psl.eu

[c] Dr. E. Izquierdo-García, Prof. V. Marchán
Departament de Química Inorgànica i Orgànica, Secció de Química Orgànica, Universitat de Barcelona (UB)
Institut de Biomedicina de la Universitat de Barcelona (IBUB), Martí i Franquès 1-11, E-08028 Barcelona, Spain.

[d] Prof. G. J. L. Bernardes,
GiMM - Gulbenkian Institute for Molecular Medicine, Avenida Prof. Egas Moniz, Lisboa, 1649-035 Portugal
Translational Chemical Biology Group, Spanish National Cancer Research Centre 26 (CNIO), Madrid 28029.

[†] These authors contributed equally to this work. Supporting information for this article is given via a link at the end of the document.

Abstract: Carbon monoxide (CO) has shown therapeutic potential across various diseases, including cancer. To enable controlled delivery, many CO-releasing molecules (CORMs) have been developed. However, their clinical translation has been limited due to concerns about stability, potential toxicity, and insufficient targeting ability. In this study, we report the synthesis and characterization of an enzyme-triggered CO-releasing molecule (**ET-CORM**) that can be site-specifically conjugated to antibodies. This novel **ET-CORM** is built on a biocompatible iron core, and releases CO upon cleavage by the cancer-associated protease cathepsin B (CatB). The incorporation of a bioorthogonal handle into **ET-CORM** enabled its efficient and site-specific conjugation to the clinically used antibody trastuzumab via the use of the interchain disulfide bonds. The resulting ET-CORM–antibody conjugate (**ET-CORM-Ab**) exhibited an average drug-to-antibody ratio (DAR) of 6.8, corresponding to approximately 20 CO molecules per conjugate. This construct allowed for selective intracellular CO delivery to HER2-overexpressing and CatB-expressing cells *in vitro*. This study represents a metal-based CORM–antibody conjugate activated by a tumor-associated enzymatic trigger, opening new avenues for investigating CO-mediated effects and advancing CO-based cancer therapies to the clinics.

Table of Contents

Table of Contents 2
1. *Supporting Figures* 3
2. *Experimental – general remarks* 11
3. *Synthetic procedures* 12
Compound 3 ((*E*)-3-(2-(2-(4-Oxo-4-phenylbut-2-enamido)ethoxy)ethoxy)propanoic acid) 12
Compound 4 (Alloc-Val-Cit-PAB-Cl) 13
Compound 6 (Alloc-Val-Cit-PAB-η^4-oxycyclohexadiene–$Fe(CO)_3$) 14
Compound 7 (H-Val-Cit-PAB-η^4-oxycyclohexadiene–$Fe(CO)_3$) 15
ET-CORM 16
ET-CORM–NAC conjugate 17
4. *CatB cleavage assay* 17
4.1. **UPLC-MS analysis** 17
4.2. **CO detection by turn-on fluorescent CO probe** 17
5. *Bioconjugation reactions* 18
5.1. **ET-CORM–Ab conjugate** 18
5.2. **Bioconjugate stability** 18
6. *Cell experiments.* 18
6.1. **Cell viability assays** 18
6.2. **Cell imaging** 19
6.3. **Flow cytometry: ET-CORM–Ab binding to the receptor using secondary antibody control** 19
7. *NMR spectra* 21
8. *Protein LC-MS spectra* 26
LC-MS of Unconjugated Trastuzumab 26
LC-MS of **ET-CORM–Ab** 27
9. *Uncropped SDS-PAGE* 29
10. *References* 30

1. Supporting Figures

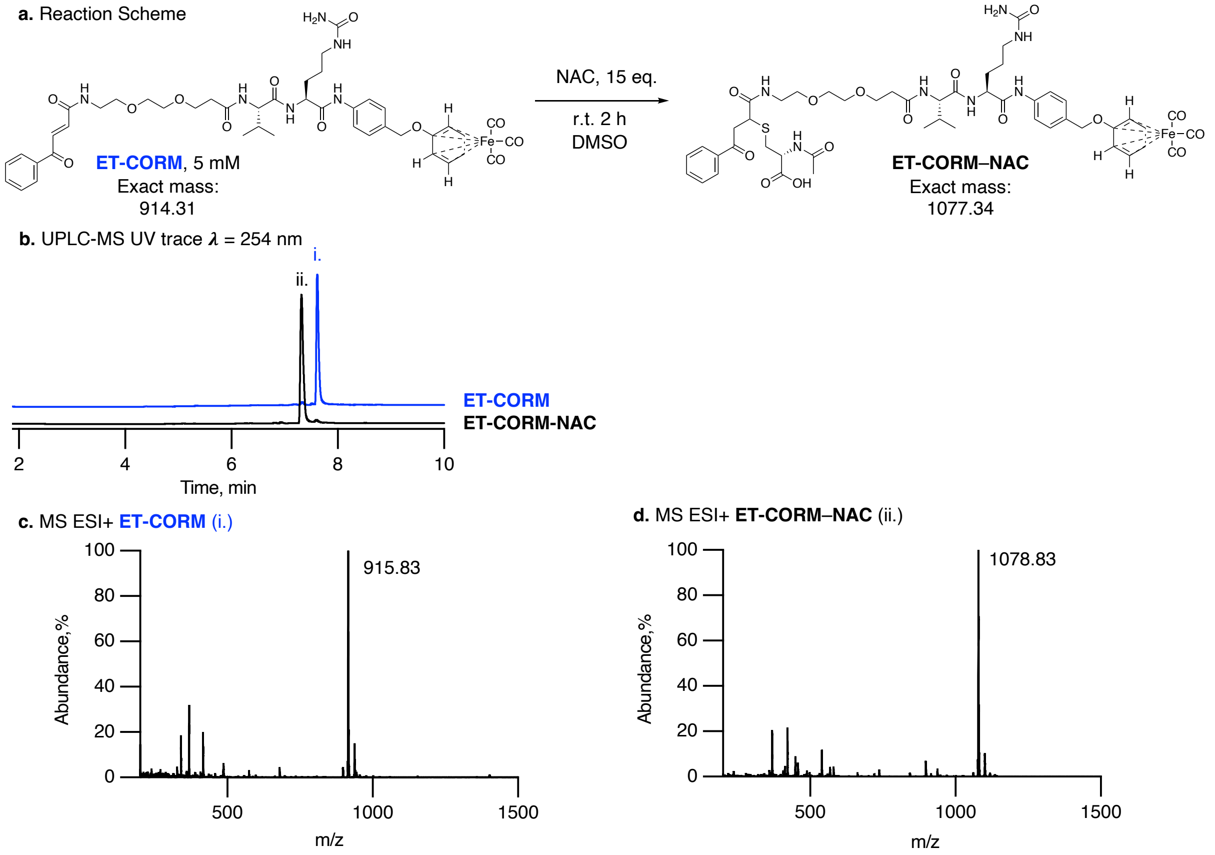

Figure S 1 Preparation and characterization of the **ET-CORM–NAC** model construct for cathepsin B cleavage studies. **a**. Reaction scheme and experimental conditions for the conjugation of **ET-CORM** with *N*-acetylcysteine (NAC); **b.** UPLC chromatograms; **c.** ESI$^+$ mass spectrum of **ET-CORM**; **d.** ESI$^+$ mass spectrum of the resulting **ET-CORM–NAC** conjugate.

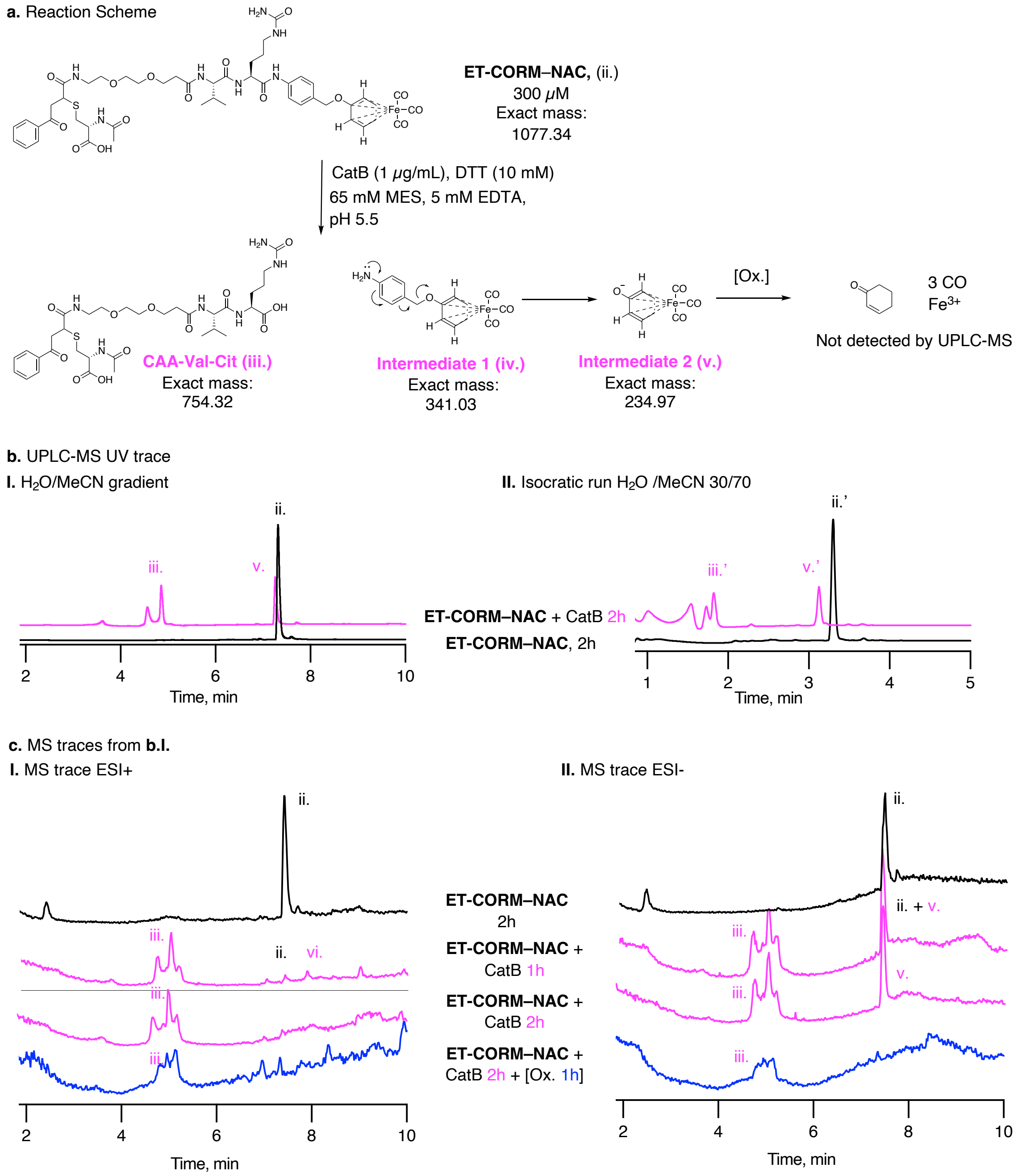

Figure S 2 Study of the proteolytic cleavage of **ET-CORM–NAC** by Cathepsin B. **a.** Reaction scheme and experimental conditions illustrating the cascade initiated by the hydrolysis of **ET-CORM–NAC**, ultimately leading to CO release. Cleavage at the **Val-Cit–PAB** site by CatB generates the *N*-acetylcysteine–carbonyl acrylic acid–valine–citrulline fragment (**CAA–Val–Cit, iii.**) along with **Intermediate 1 (iv.)**. As previously reported in the literature,[1] **Intermediate 1** is highly unstable and rapidly undergoes self-immolation to form **Intermediate 2 (v.)**. **Intermediate 2** is expected to release three molecules of CO following oxidation under mild conditions. **b.I.** UPLC-MS UV trace (λ = 254 nm) of **ET-CORM–NAC** after 2 hours incubation in the presence (pink) and in the absence (black) of CatB enzyme. Peaks **ii** and **v** elute at similar retention times under a linear H_2O–ACN gradient (0.1% FA, inlet method B, see below); however, they can be successfully resolved using an isocratic 3:7 H_2O–ACN (0.1% FA) method. (**b.II.**). **c.** ESI-MS positive mode trace spectra of **ET-CORM–NAC (ii.)** incubated at 37 °C with and without CatB enzyme for 1 hour, 2 hours, and an additional hour after the addition of an oxidant NaClO (2 eq. of dithiothreitol (DTT)) to quench the excess of DTT, which is required to maintain the activity of commercially sourced recombinant CatB. The formation of **Intermediate 2** was observed only in the presence of CatB, and the corresponding signal disappeared following oxidant addition. For mass spectra see Figure S3. This reflects the formation of iron(III) ions and the release of CO, consistent with previously reported mechanisms.[1] MES buffer - 2-(*N*-morpholino)ethanesulfonic acid buffer, EDTA - ethylenediaminetetraacetic acid.

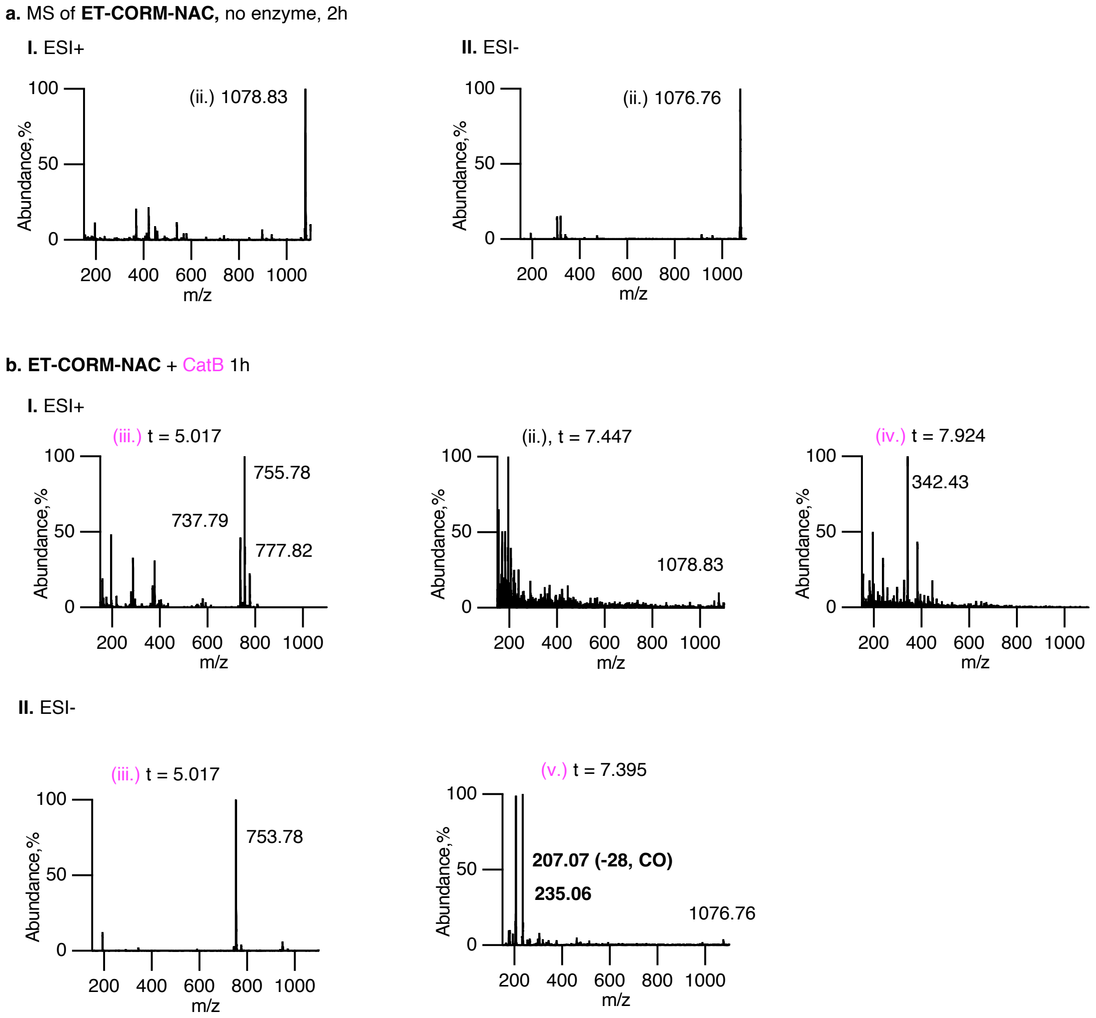

Figure S 3 ESI-MS spectra of the representative peaks detected in the MS traces from Figure S2, with key species identified by their corresponding m/z values. Major ions are labelled, and the relevant compound structures are shown in Figure S2. **a. ET-CORM–NAC (ii.)** remains stable under the reaction conditions in the absence of Cathepsin B (CatB) enzyme, as demonstrated by both ESI$^+$ and ESI$^-$ modes. **b.** ESI$^+$ and ESI$^-$ signal integration at representative retention times after 1 hour of **ET-CORM–NAC** incubation with CatB shows a clear mass corresponding to the **CAA-Val-Cit** fragment (**iii.**) in both modes (t = 5.017 min). In contrast, only trace of the **ET-CORM–NAC** parent compound is detected (t = 7.747 min, ESI$^+$ and ESI$^-$), alongside a minor signal corresponding to **Intermediate 1 (iv.).** After 2 hours of incubation with CatB, both the **ET-CORM–NAC (ii.)** and **Intermediate 1 (iv.)** signals are no longer detectable, as shown in the MS trace (Figure S2c), indicating complete enzymatic cleavage. Notably, **Intermediate 2 (v.)** is exclusively detected in ESI$^-$ mode, with a primary signal at m/z 235. A secondary peak at m/z 207 is also observed, consistent with the loss of one carbon monoxide molecule (Δm = 28).

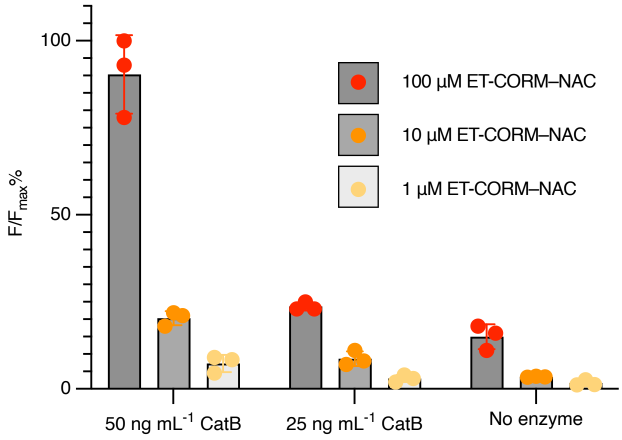

Figure S 4 The fluorescence turn-on of the CO-sensitive probe **1-Ac** after 24 h incubation with the ET-CORM–NAC conjugate was dependent on both the concentration of the enzyme and that of the conjugate, n = 3. [**1-Ac**] = 5 µM in MES buffer (65 mM MES, 5 mM EDTA, 0.5 mM DTT, pH 5.5).

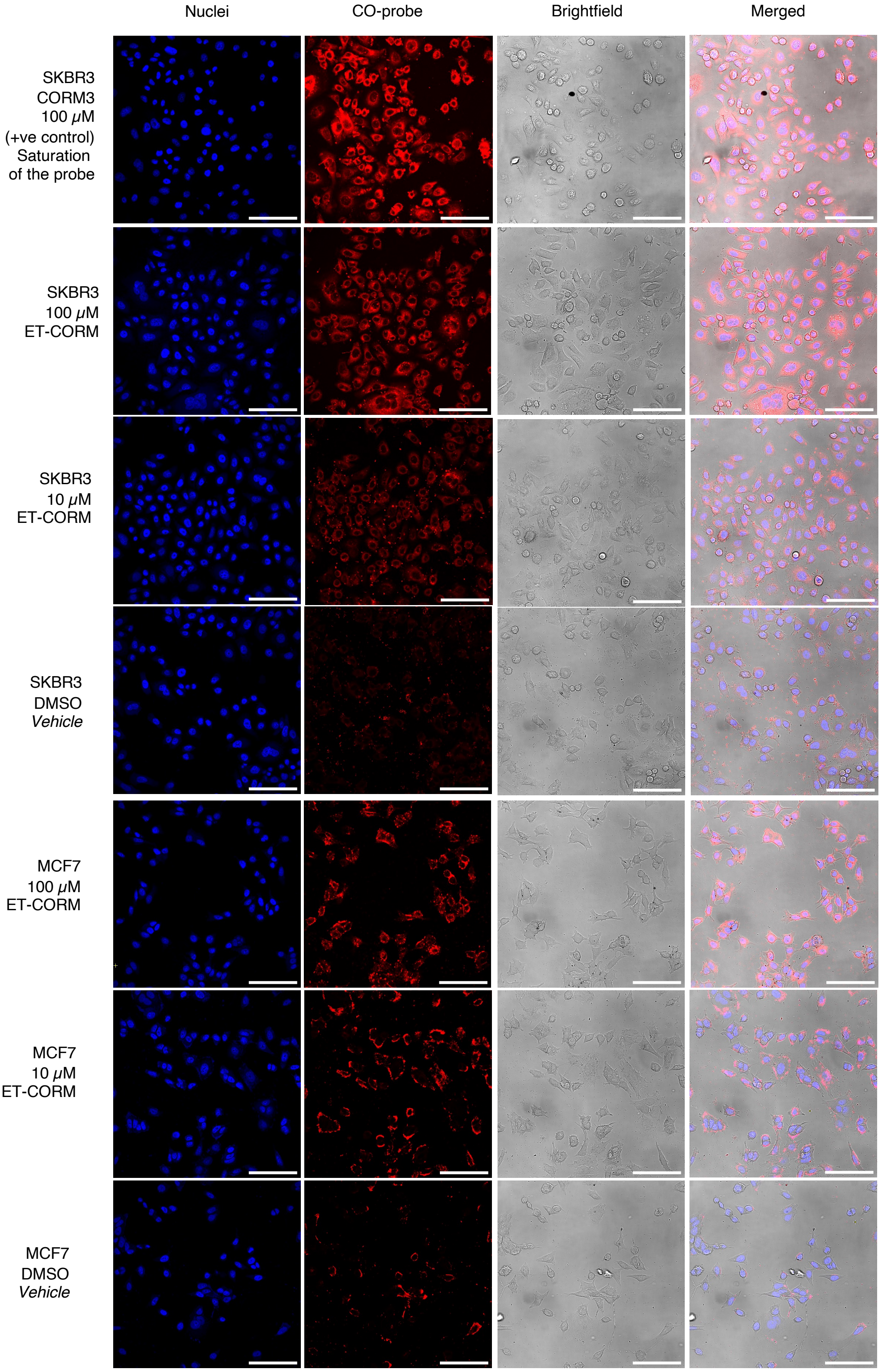

Figure S 5 More detail representation of Figure 2b in the main text. Quantification of extensive combined images provided in Figure S9a. Confocal microscopy images showing cellular CO release triggered by endogenous cathepsin B in untreated and treated SKBR3 and MCF7 cells (100 μM or 10 μM). After an initial 30-minute pre-treatment with 5 μM **1-Ac** CO probe, either **ET-CORM**, vehicle (DMSO) or the positive control CORM3 was added. Following 60 minutes of incubation, cells were fixed and imaged. An increase in fluorescence represents the turn-on response of the **1-Ac** CO probe (λ_{ex} = 561 nm, λ_{em} = 570–620 nm). Scale bar represents 100 μm. N.B. According to the Human Protein Atlas (data available from v24.proteinatlas.org, https://www.proteinatlas.org/ENSG00000164733-

CTSB/cell+line, accessed 2025.08.20, ref. 56 in the main text), CatB RNA levels for SKBR3 and MCF7 cell lines are 93.2, and 90.8, respectively, hence, similar level of fluorescence is expected.

a. Fluorescence microscopy images

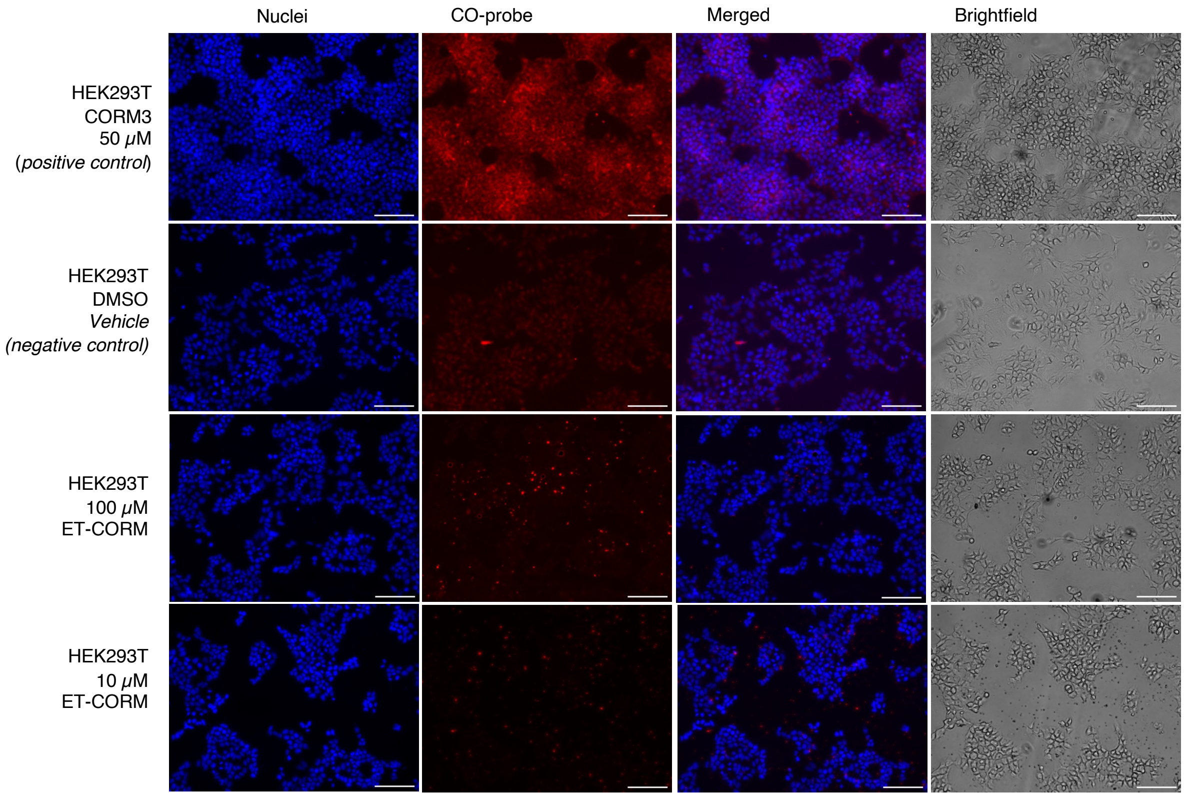

b. Fluorescence quantification

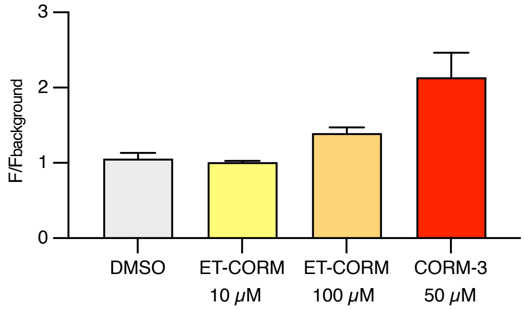

Figure S 6 a. Fluorescence microscopy images showing cellular CO release triggered by endogenous cathepsin B in untreated and treated HER2-low HEK293T cells. After an initial 30-minute pre-treatment with 5 μM **1-Ac** CO probe, either **ET-CORM** (100 μM or 10 μM), vehicle (DMSO), or the positive control (CORM3) was added. After 60 minutes of incubation, cells were fixed and imaged. An increase in fluorescence represents the turn-on response of the **1-Ac** CO probe, which was observed in the positive control (CORM3), while the response to ET-CORM in the HEK293T cell line was sluggish. Scale bar represents 100 μm. **b.** Quantification of **1-Ac** fluorescence in the images shown in (**a.**) and additional biological replicates Data is presented as median of fluorescence per image taken ± SD (*n* = 9). N.B. According to the Human Protein Atlas (data available from v24.proteinatlas.org, https://www.proteinatlas.org/ENSG00000164733-CTSB/cell+line, accessed 2025.08.20, ref. 56 in the main text), CatB RNA levels for HEK293T, SKBR3, and MCF7 cell lines are 49.4, 93.2, and 90.8, respectively. Therefore, little to no turn-on fluorescence signal of CO probe is expected in HEK293T treated with ET-CORM.

a. ET-CORM–Ab at t = 0 h

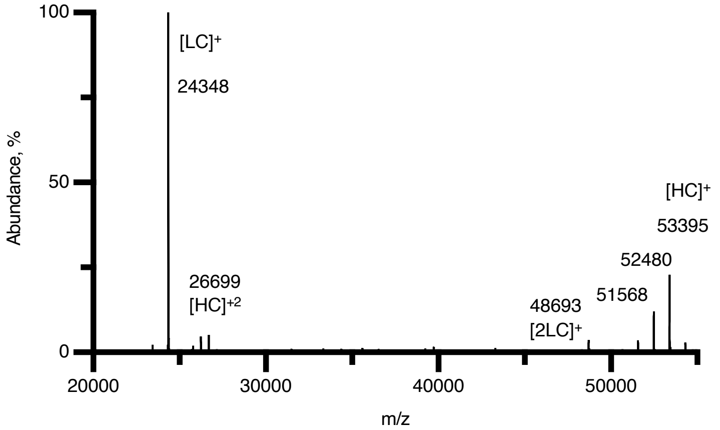

b. ET-CORM–Ab stability in PBS (pH 7.4) for 48h by MS

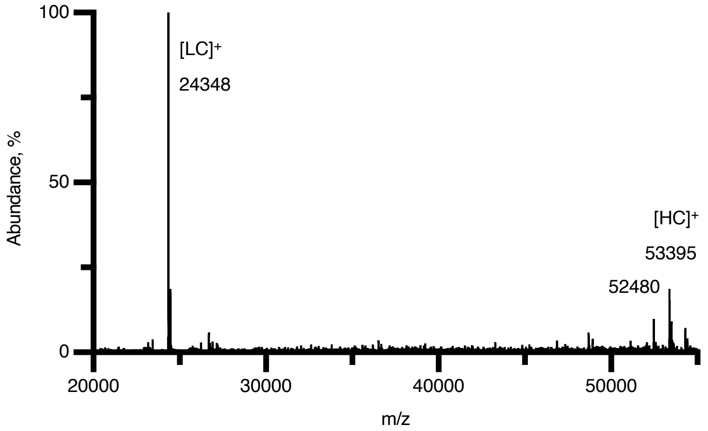

Figure S 7 The **ET-CORM–Ab** conjugate did not spontaneously release CO in PBS over a 48-hour incubation at 37 °C, as confirmed by the absence of m/z changes in mass spectrometry (MS) analysis. Spontaneous CO release would result in the mass reduction in both heavy and light chains; no hydrolysis of conjugation handle from the protein was observed either. **a.** t = 0, or **b.** endpoint in PBS. PBS – phosphate-buffered saline.

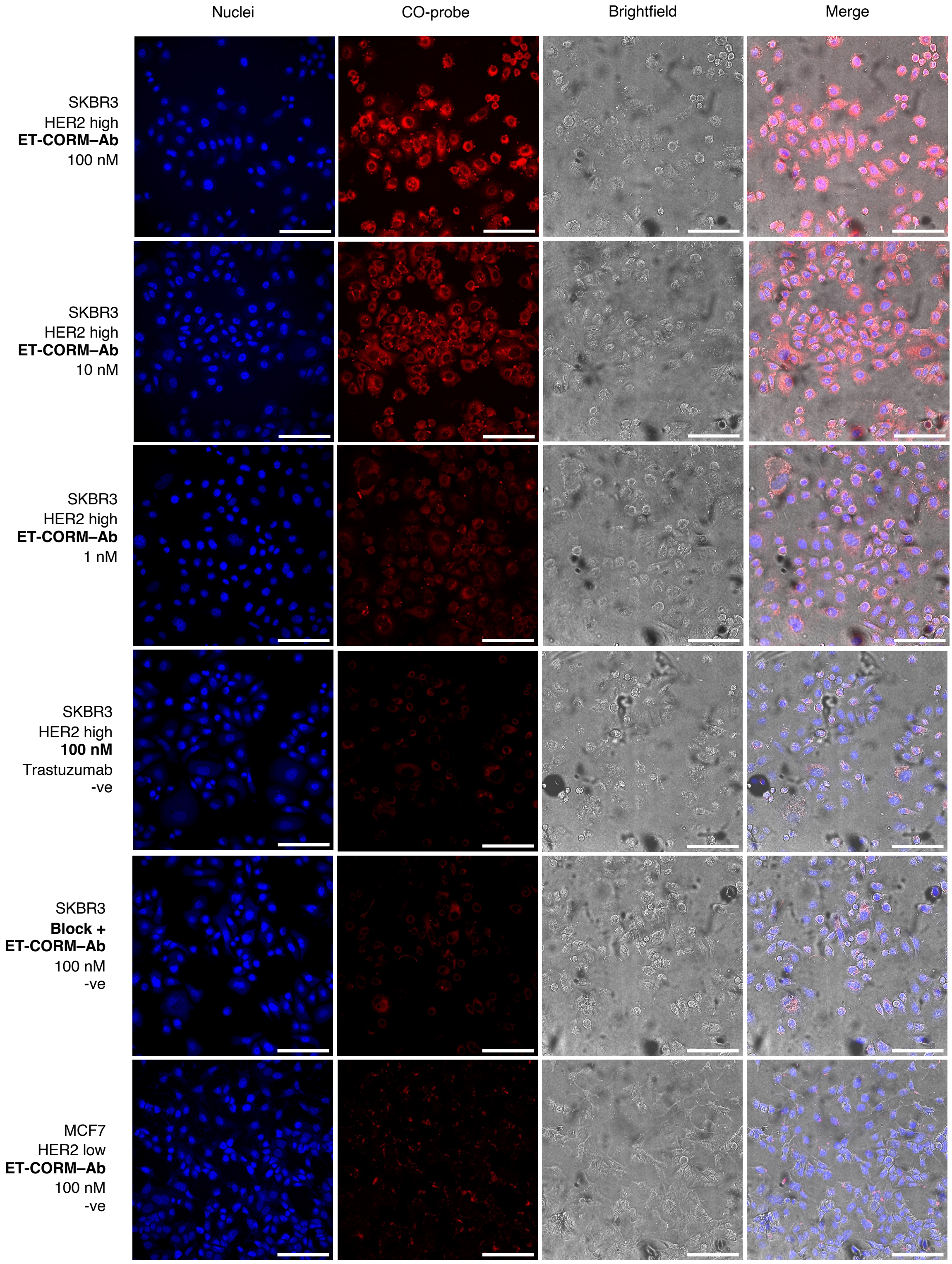

Figure S 8 More detail representation of Figure 4 in the main text. Quantification of extensive combined images provided in Figure S9b. Confocal microscopy images showing cellular CO release in treated SKBR3 (HER2-high) and MCF7 (HER2-low) cells with **ET-CORM–Ab**. After an initial 30-minute treatment with 5 μM **1-Ac** CO probe, either **ET-CORM–Ab** or control treatment was added. Following 2 hours of incubation, cells were fixed and imaged. DAPI was used to stain nuclei (blue), and intracellular CO release was visualized using the turn-on **1-Ac** CO probe (red, λ_{ex} = 561 nm, λ_{em} = 570–620 nm). The white scale bar represents 100 μm. Pre-treatment of SKBR3 cells with non-fluorescent trastuzumab (i.e., HER2 receptor blockade) abolished the fluorescence response following 100 nM **ET-CORM–Ab** incubation.

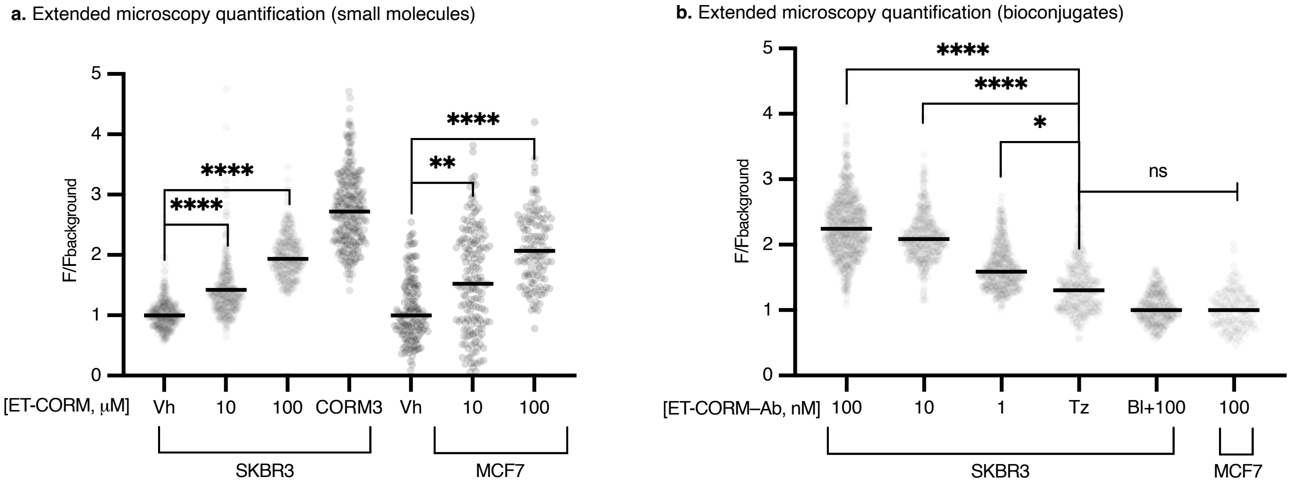

Figure S 9 Extended fluorescence quantification from images in figures S5 and S8, for **a.** and **b.**, respectively. **a.** Turn-on fluorescence of the **1-Ac** CO probe was significant when both SKBR3 and MCF7 cells were treated with 10 μM and 100 μM **ET-CORM**, compared to vehicle (Vh) control (DMSO), while the turn-on response for positive control CORM3 was even higher. **b.** Turn-on fluorescence of the **1-Ac** CO probe was significant in HER2-high SKBR3 cells treated with **ET-CORM–Ab** at concentrations ranging from 100 nM to 1 nM, while no significant fluorescence increase was observed in HER2-low MCF7 cells. Pre-treatment of SKBR3 cells with non-fluorescent trastuzumab (i.e., HER2 receptor blockade) effectively blocked the fluorescence response observed after incubation with 100 nM **ET-CORM–Ab**. Background normalization was performed relative to the median fluorescence of the vehicle control. Statistically significant differences were determined using an unpaired t-test and are indicated as **** ($P < 0.00005$), ** ($P < 0.005$), * ($P < 0.05$), and ns ($P > 0.05$).

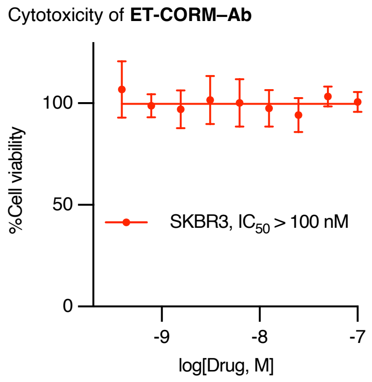

Figure S 10 Cytotoxicity studies of **ET-CORM–Ab** suggested no cytotoxicity (IC_{50} > 100 nM) to HER2-high CatB-expressing cell line SKBR3 in 72-hour assay. Data represent three independent experiments.

2. Experimental – general remarks

Unless otherwise stated, common chemicals and solvents (HPLC grade or reagent grade quality) were purchased from commercial sources and used without further purification. Precursors **2**[2] and **5**[3] were prepared according to previously reported procedures. Anhydrous THF was freshly distilled from sodium under a nitrogen atmosphere, and all moisture-sensitive reactions were conducted under strictly inert conditions using standard Schlenk techniques. A hot plate magnetic stirrer, together with an aluminum reaction block of the appropriate size, was used as the heating source in all reactions requiring heat. Aluminum plates coated with a 0.2 mm thick layer of silica gel 60 F_{254} were used for thin-layer chromatography analyses (TLC), whereas flash column chromatography purification was carried out using silica gel 60 (230-400 mesh). ^{1}H and ^{13}C NMR spectra were recorded at 25 °C on Bruker Avance III HD 400 MHz or Bruker Avance Neo 500 MHz spectrometers, using the residual solvent signal as the internal chemical shift reference. Chemical shifts are reported in part per million (ppm) in the δ scale, coupling constants in Hz and multiplicity as follows: s (singlet), d (doublet), t (triplet), q (quartet), qt (quintuplet), m (multiplet), dd (doublet of doublets), dt (doublet of triplets), td (triplet of doublets), br (broad signal), etc. Electrospray ionization mass spectra (ESI-MS) were recorded on a QTOF Agilent 6560 mass spectrometer equipped with ionic mobility cell, coupled to Agilent 1290 Infinity II UHPLC chromatograph. Reversed-phase HPLC analyses were carried out on a Waters Alliance 2795 chromatograph with a DAD detector using a Jupiter Proteo C12 column (150 × 4.6 mm, 90 Å, 4 µm, flow rate: 1 mL/min) using linear gradients of 0.1% formic acid in Milli-Q H_2O (A) and 0.1% formic acid in ACN (B).

Data analysis. GraphPad Prism v10.14.1 was used to generate graphs and for statistical student t-test and ANOVA analysis. Data sets with a Gaussian distribution were analyzed by parametric unpaired t test and data sets that did not pass the normality tests were analyzed by nonparametric unpaired Mann–Whitney test. Clearance data sets were analyzed using Fisher's exact test. All were two-sided tests with a confidence interval of 95%.
Fiji ImageJ2 (v2.14.0/1.54f) was used to analyze and quantify SDS-PAGE gels and quantify mean fluorescence per cell in microscopy images, where acceptable. Cartoon representations were created with *BioRender*.

SDS-PAGE was done with NuPAGE™ Bis-Tris Mini Protein Gels, 4–12%, 1 .0–1.5 mm and NuPAGE™ MES SDS Running Buffer (20X) diluted in Milli-Q water, at a constant voltage of 200 V and using Invitrogen™ SeeBlue™ Plus2 Pre-stained Protein Standard and stained with Instant Blue™ Coomassie protein stain. Gels were imaged using Bio-Rad ChemiDoc. and quantified, when required, using Fiji ImageJ2 (version 2.14.0/1.54f).

UPLC-MS: Mass spectrometry of protein and small molecule samples. LC–MS analysis of protein samples was carried out using a Waters SQD2 mass spectrometer using inlet method A for protein and B for peptides, in combination with an Acquity UPLC system with an Acquity UPLC BEH300 C4 column (130 Å1.7 µm, 2.1 × 50 mm) for proteins or an Acquity UPLC BEH C18 column (130 Å1.7 µm, 2.1 × 50 mm) for peptides and small molecules. The SQD2 mass spectrometer mobile phase consisted of solvent A (0.1% formic acid in Milli-Q water), solvent B (0.1% formic acid in ACN). Gradient methods were as follows: inlet method A: 5% to 72% B in 6 min, then 72% B for 1.5 min, followed by a gradient from 72% to 5% B over 0.25 mins and finally, 5% B for 1.25 mins. Inlet method B: 5% B for 0.5 mins, followed by a gradient from 5% to 90% B over 5.5 mins, then 90% B for 2.5 min, followed by a gradient from 90% to 5% B over 0.25 min and finally, 5% B for 3.25 min. The capillary voltage of the electrospray source for the Waters SQD2 mass spectrometer was 3.0 kV with a cone voltage of 30 V and the desolvation gas used was nitrogen, with a flow rate of 800 L h−1. The ion series was obtained through integration of the

major peaks of the chromatogram. Following this, the total mass spectra were reconstructed using the MaxEnt1 algorithm on the MassLynx software (v. 4.1), according to manufacturer's guidelines.

3. Synthetic procedures

Compound 3 ((*E*)-3-(2-(2-(4-Oxo-4-phenylbut-2-enamido)ethoxy)ethoxy)propanoic acid)

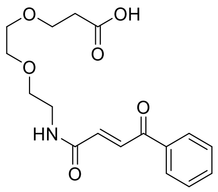

H_2N-PEG$_2$-COOtBu (150 mg, 0.64 mmol, 1.0 equiv) was dissolved in a 1:1 mixture of TFA-CH_2Cl_2 (2 mL), and the solution was stirred at room temperature for 4 h. Upon reaction completion, the volatiles were removed under reduced pressure, and the residue was co-evaporated with CH_2Cl_2 (3 × 10 mL) to remove residual TFA. The resulting crude carboxylic acid **1** (110 mg, 0.62 mmol, 1.0 equiv) was dissolved in anhydrous CH_2Cl_2 (10 mL), and triethylamine (NEt_3, 430 µL, 3.1 mmol, 5.0 equiv) was added, followed by the portionwise addition of the activated ester **2**[2] (210 mg, 0.62 mmol, 1.0 equiv). After stirring overnight at room temperature, the reaction mixture was concentrated under reduced pressure. The residue was suspended in water (25 mL), and the aqueous phase was acidified to pH 2 with 1.0 M HCl, and extracted with CH_2Cl_2 (3 × 25 mL). The combined organic layers were dried over anhydrous $MgSO_4$, filtered, and concentrated under reduced pressure, and the crude product was purified by flash chromatography on silica gel (0–10% MeOH in CH_2Cl_2) to afford compound **3** as an off-white solid (162 mg, 78%).

^{1}H NMR (500 MHz, CD_3OD) δ 8.03 (dt, J = 7.1, 1.3 Hz, 2H), 7.88 (d, J = 15.3 Hz, 1H), 7.73 – 7.62 (m, 1H), 7.55 (t, J = 7.8 Hz, 2H), 7.05 (d, J = 15.3 Hz, 1H), 3.75 (t, J = 6.2 Hz, 2H), 3.64 – 3.59 (m, 6H), 3.51 (t, J = 5.4 Hz, 2H), 2.55 (t, J = 6.2 Hz, 2H).
^{13}C{^{1}H}NMR (126 MHz, CD_3OD) δ 191.5, 175.3, 166.7, 138.3, 136.6, 134.9, 134.1, 130.0, 129.8, 71.4, 71.2, 70.3, 67.8, 40.9, 35.7.
HRMS (ESI): m/z $[M+H]^+$ calcd. for $C_{17}H_{21}NO_6H$: 336.1442; found: 336.1443

Compound 4 (Alloc-Val-Cit-PAB-Cl)

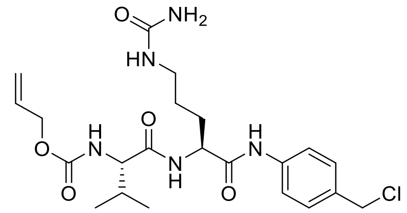

Alloc-Val-Cit-PAB-OH (200 mg, 0.43 mmol, 1 equiv) was suspended in anhydrous THF (20 mL) under an Ar atmosphere. Thionyl chloride (55 μL, 0.76 mmol, 1.75 equiv) was added dropwise, and the reaction mixture was stirred at room temperature for 4 h. Excess thionyl chloride was quenched by adding a few drops of MeOH, and the volatiles were removed under reduced pressure at 35 ºC. The crude compound **4**, an off-white solid (203 mg, 98% yield), was used directly in the subsequent reaction without further purification.

^{1}H NMR (400 MHz, DMSO-d_6) δ 10.09 (s, 1H), 8.10 (d, J = 7.5 Hz, 1H), 7.60 (d, J = 8.3 Hz, 2H), 7.36 (d, J = 8.3 Hz, 2H), 7.24 (d, J = 8.8 Hz, 1H), 5.97 (t, J = 6.0 Hz, 1H), 5.95 – 5.80 (m, 1H), 5.41 (s, 2H), 5.30 (d, J = 16.3 Hz, 1H), 5.17 (d, J = 9.9 Hz, 1H), 4.71 (s, 2H), 4.48 (d, J = 4.6 Hz, 2H), 4.41 (q, J = 7.2 Hz, 1H), 3.95 – 3.86 (m, 1H), 3.09 – 2.99 (m, 1H), 2.99 – 2.89 (m, 1H), 2.04 – 1.91 (m, 1H), 1.74 – 1.64 (m, 1H), 1.64 – 1.55 (m, 1H), 1.50 – 1.38 (m, 2H), 0.88 (d, J = 6.6 Hz, 3H), 0.83 (d, J = 6.7 Hz, 3H).
^{13}C{^{1}H} NMR (101 MHz, DMSO-d_6) δ 171.3, 170.7, 158.9, 156.0, 139.0, 133.6, 132.4, 129.5, 119.1, 116.9, 64.4, 60.0, 53.1, 46.2, 38.5, 30.4, 29.4, 26.8, 19.2, 18.1.
HRMS (ESI): m/z $[M+H]^+$ calcd. for $C_{22}H_{32}ClN_5O_5H$: 482.2165; found: 482.2162.

Compound 6 (Alloc-Val-Cit-PAB-η^4-oxycyclohexadiene–$Fe(CO)_3$)

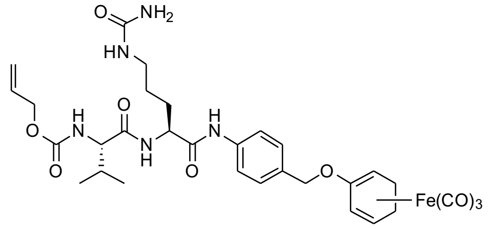

Complex **5**[3] (108 mg, 0.27 mmol, 1.1 equiv) was dissolved in anhydrous THF (10 mL) under an Ar atmosphere, and the solution was cooled to 0 ºC. Sodium hydride (60% w/w dispersion in mineral oil, 12 mg, 0.30 mmol, 1.2 equiv) and TBAF (1 M in THF, 300 µL, 0.30 mmol, 1.2 equiv) were added sequentially, and the mixture was stirred for 10 minutes at 0 ºC. Compound **4** (120 mg, 0.25 mmol, 1 equiv) was then added as a solution in anhydrous THF (5 mL), the ice bath was removed, and the reaction mixture was stirred overnight at room temperature. Unreacted NaH was quenched with a few drops of MeOH, and the volatiles were removed under reduced pressure at 35 ºC. Purification of the crude residue by flash chromatography on silica gel (0-6% MeOH in DCM) yielded compound **6** as a white solid (75 mg, 44%).

^{1}H NMR (500 MHz, CD_3OD) δ 7.60 (d, *J* = 8.6 Hz, 2H), 7.34 (d, *J* = 8.6 Hz, 2H), 5.94 (ddt, *J* = 16.2, 10.6, 5.4 Hz, 1H), 5.43 – 5.36 (m, 1H), 5.36 – 5.26 (m, 1H), 5.18 (dq, *J* = 10.5, 1.4 Hz, 1H), 4.95 (d, *J* = 11.1 Hz, 1H), 4.69 (d, *J* = 11.0 Hz, 1H), 4.60 – 4.44 (m, 3H), 3.97 (d, *J* = 6.8 Hz, 1H), 3.58 – 3.43 (m, 1H), 3.26 – 3.15 (m, 1H), 3.11 (dt, *J* = 13.5, 6.7 Hz, 1H), 2.84 (ddd, *J* = 6.7, 3.6, 2.3 Hz, 1H), 2.08 (dq, *J* = 14.6, 7.9, 7.3 Hz, 1H), 1.90 (dd, *J* = 13.6, 6.2 Hz, 1H), 1.77 – 1.70 (m, 3H), 1.63 – 1.50 (m, 4H), 0.98 (d, *J* = 6.8 Hz, 3H), 0.96 (d, *J* = 6.7 Hz, 3H).
^{13}C{^{1}H} NMR (126 MHz, CD_3OD) δ 213.0, 172.3, 162.4, 140.5, 139.6, 133.4, 129.9, 121.1, 70.3, 60.8, 56.7, 54.9, 52.7, 40.1, 30.7, 29.8, 27.9, 25.8, 24.5, 19.5, 17.8.

HRMS (ESI): *m/z* $[M+H]^+$ calcd. for $C_{31}H_{39}FeN_5O_9H$: 682.2170; found: 682.2196.

Compound 7 (H-Val-Cit-PAB-η^4-oxycyclohexadiene–$Fe(CO)_3$)

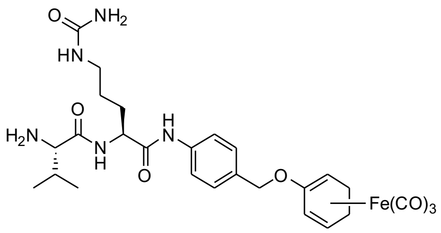

To a stirred solution of compound **6** (67 mg, 0.10 mmol, 1 equiv) in anhydrous THF (10 mL) under an Ar atmosphere were sequentially added tetrakis(triphenylphosphine)palladium(0) (11 mg, 10 mol%) and diethylamine (52 µL, 0.49 mmol, 5 equiv), and the reaction mixture was stirred overnight at room temperature. The mixture was concentrated *in vacuo* and the residue was purified by flash chromatography on silica gel (10-40% MeOH in DCM) to give compound **7** as a white solid (40 mg, 68%).

^{1}H NMR (400 MHz, CD_3OD) δ 7.59 (d, J = 8.5 Hz, 2H), 7.34 (d, J = 8.6 Hz, 2H), 5.38 (dd, J = 6.9, 2.3 Hz, 1H), 4.95 (d, J = 11.1 Hz, 1H), 4.68 (d, J = 11.0 Hz, 1H), 4.63 – 4.50 (m, 1H), 3.50 (p, J = 2.7 Hz, 1H), 3.36 – 3.34 (m, 1H), 3.25 – 3.08 (m, 2H), 2.84 (ddd, J = 8.9, 4.5, 2.7 Hz, 1H), 2.07 (dt, J = 13.3, 6.7 Hz, 1H), 1.95 – 1.83 (m, 1H), 1.83 – 1.46 (m, 7H), 0.98 (dd, J = 20.3, 6.9 Hz, 6H).
^{13}C{^{1}H} NMR(126 MHz, CD_3OD) δ 213.0, 172.3, 162.4, 140.5, 139.6, 133.4, 129.9, 121.1, 70.3, 60.8, 56.7, 54.9, 52.7, 49.0, 40.1, 32.8, 30.7, 29.8, 28.7, 27.9, 25.8, 24.5, 19.5, 17.8.
HRMS (ESI): m/z $[M+H]^+$ calcd. for $C_{27}H_{35}FeN_5O_7H$: 598.1959; Found: 598.1981.

ET-CORM

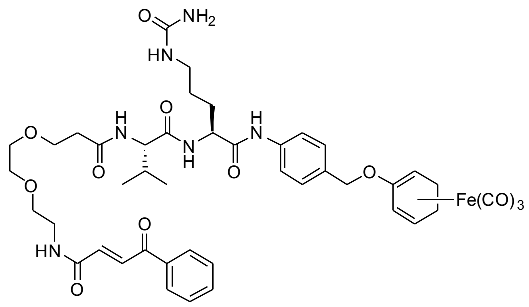

To an ice-cooled, stirred solution of compound **3** (27 mg, 80 µmol, 1.2 equiv) in anhydrous DMF (4 mL) under an argon atmosphere, HATU (33 mg, 87 µmol, 1.3 equiv) and DIPEA (35 µL, 201 µmol, 3.0 equiv) were sequentially added, and the mixture was stirred at 0 °C for 15 minutes. Subsequently, a solution of compound **7** (40 mg, 66 µmol, 1.0 equiv) in anhydrous DMF was added dropwise while maintaining the temperature at 0 °C. The reaction was then gradually allowed to warm to room temperature and furhter stirred for 4 h. The mixture was concentrated *in vacuo* and the residue was purified by reverse-phase HPLC (50-100% B in A. A: H_2O, 0.1% HCOOH; B: ACN, 0.1% HCOOH) to give **ET-CORM** as a white solid (23 mg, 37%).

^{1}H NMR (400 MHz, CD_3OD) δ 8.13 – 7.97 (m, 2H), 7.89 (d, J = 15.3 Hz, 1H), 7.70 – 7.61 (m, 1H), 7.62 – 7.58 (m, 2H), 7.58 – 7.47 (m, 2H), 7.32 (d, J = 8.6 Hz, 2H), 7.09 (d, J = 15.3 Hz, 1H), 5.38 (ddd, J = 6.7, 2.3, 0.8 Hz, 1H), 4.93 (d, J = 11.1 Hz, 1H), 4.67 (d, J = 11.1 Hz, 1H), 4.51 (dd, J = 9.0, 5.1 Hz, 1H), 4.29 – 4.20 (m, 1H), 3.76 (t, J = 6.0 Hz, 2H), 3.65 – 3.55 (m, 6H), 3.57 – 3.45 (m, 3H), 3.19 (dt, J = 13.7, 6.9 Hz, 1H), 3.10 (dt, J = 13.4, 6.6 Hz, 1H), 2.83 (ddd, J = 6.7, 3.6, 2.4 Hz, 1H), 2.55 (t, J = 5.9 Hz, 2H), 2.10 (h, J = 6.8 Hz, 1H), 1.99 – 1.84 (m, 1H), 1.81 – 1.67 (m, 3H), 1.63 – 1.44 (m, 2H), 0.98 (dd, J = 6.8, 5.6 Hz, 6H).
^{13}C{^{1}H} NMR (101 MHz, CD_3OD) δ 212.9, 191.5, 174.3, 173.9, 172.3, 166.7, 162.3, 140.4, 139.6, 138.3, 136.7, 134.9, 134.0, 133.3, 130.0, 129.9, 129.8, 121.2, 71.3, 70.3, 70.2, 68.2, 60.5, 56.7, 55.0, 52.7, 40.9, 40.3, 37.3, 31.9, 30.8, 30.4, 27.9, 25.8, 24.5, 19.8, 18.8.
HRMS (ESI): *m/z* $[M+H]^+$ calcd. for $C_{44}H_{54}FeN_6O_{12}H$: 915.3222; Found: 915.3232.

ET-CORM–NAC conjugate

ET-CORM was dissolved in DMSO at a concentration of 20 mM, and 40 µL of this solution was mixed with 120 µL of *N*-acetylcysteine (100 mM, 15 equivalents). UPLC-MS analysis confirmed complete conversion to **ET-CORM–NAC** after 2 hours.

4. CatB cleavage assay

4.1. UPLC-MS analysis

ET-CORM–NAC stock solution (5 mM in biological grade DMSO, 6 µL) was diluted in MES buffer (30 µL 65 mM MES, 5 mM EDTA, pH 5.5). In a separate vial, 5 µL of a freshly prepared DTT stock solution (200 mM in Milli-Q water) was added to 1 µL of Cathepsin B stock solution (0.1 µg µL^{-1}, Merck 219362-50µg) and incubated for 5 minutes to activate the enzyme. Then, the solution of **ET-CORM–NAC** in MES buffer and the activated Cathepsin B solution were combined, and the reaction was incubated at 37°C. At desired timepoints, 10 µL aliquots were removed and flash frozen for further analysis via UPLC-MS. For the negative control reaction, 1 µL of assay buffer was added instead of the cathepsin B enzyme stock solution. Prior to UPLC-MS analysis, 10 µL of ACN was added to each aliquot to precipitate the enzyme. The samples were vortexed and centrifuged at 14,000 g for 2 minutes, and the resulting supernatant was injected into the UPLC-MS system. The reaction was performed in duplicate with the recorded ESI-MS ES+ and ESI- spectra averaged.
Final reaction conditions: **ET-CORM–NAC** (300 µM), MES buffer (65 mM MES, 5 mM EDTA, 10 mM DTT, pH 5.5), Cathepsin B enzyme (1 µg mL^{-1}). Final concentration of DMSO – 6%.

4.2. CO detection by turn-on fluorescent CO probe

For CO release studies in solution, the **1-Ac** probe was synthesized following the procedure described in the literature,[4] and a 10 mM stock solution (2000×) was prepared in DMSO. Before the experiment, the stock solution was further diluted 10-fold with DMSO (to 200×) and then filtered through a sterile 0.22 µm filter. This solution was subsequently diluted 1:100 (to 2×) in MES buffer (65 mM MES, 5 mM EDTA, pH 5.5), and 100 µL of the resulting solution was added to each well of a 96-well black flat-bottom plate. **ET-CORM–NAC** solution was prepared by diluting the 5 mM stock solution in DMSO to 4x the desired final concentration (*i.e.* 100 µM, 10 µM, or 1 µM) in MES buffer. Then, 50 µL of the compound solution or vehicle control (DMSO) was added to the wells of the same 96-well black flat-bottom plate. Lastly, CatB enzyme was activated by mixing 1 µL of enzyme stock solution (0.1 µg µL^{-1}) with 5 µL of freshly prepared DTT (200 mM in Milli-Q water) and incubating for 5 minutes. The activated enzyme solution was then diluted with 494 µL of MES buffer, and 50 µL of the resulting solution was added to each designated well. Control wells (DTT only) received no enzyme. The plate was incubated at 37°C for 24 h, and fluorescence was measured with a Molecular Devices SpectraMax MiniMax 300 Imaging cytometer i3x (λ_{ex} = 561 nm, λ_{em} = 660 nm). Each condition was tested in triplicate.
Final reaction conditions: **ET-CORM–NAC** (1–100 µM), **1-Ac** probe (5 µM), MES buffer (65 mM MES, 5 mM EDTA, 0.5 mM DTT, pH 5.5), Cathepsin B enzyme (1:2000, 50 ng mL^{-1} or 1:4000, 25 ng mL^{-1}). Final concentration of DMSO – 2.5%.
Background (vehicle) control was subtracted from the sample control and the value was divided by the observed maximum fluorescence.

5. Bioconjugation reactions

All buffers for bioconjugation were prepared on site. TCEP was prepared in Milli-Q water and the pH adjusted as required. Trastuzumab was obtained from MedChemExpress (HY-P9907) and reconstituted in Milli-Q water to the concentration of 7.2 mg mL^{-1}. The concentration of proteins or their conjugates was measured with a Nanodrop™One microvolume UV spectrometer using the reported molar extinction coefficient $\varepsilon_{(trastuzumab)}$= 225000 $M^{-1}cm^{-1}$.[5] Where not acceptable (payload is UV active), the concentration was measured by Pierce™ 660nm Protein Assay Reagent (ThermofisherScientific Cat. no 22660) using trastuzumab for a standard curve.
For LC-MS analysis, IgG antibodies had to be fully reduced into heavy chain (HC) and light chain (LC). Briefly, 1 µL of the reaction mixture was diluted with 8 µL of PBS, followed by addition of DTT (1 µL, 100 mM stock), and incubated at 37ºC for 30 min.
For SDS-PAGE analysis, samples were diluted in PBS to ca. 2 µM in a final volume of 9 µL. DTT (1 µL, 100 mM stock) or PBS (1 µL, 100 mM stock) was then added for reduced and non-reduced conditions, respectively, before addition of LDS sample buffer (4x, 3.3 µL) and heating at 95ºC for 5 minutes.

5.1. ET-CORM–Ab conjugate

To 250 µL of Trastuzumab in PBS (49.5 µM, 7.2 mg/mL), TCEP (5 eq., 5.0 µL, 10 mM in water) was added, and the mixture was incubated at 37ºC for 30 min. Subsequently, **ET-CORM** stock solution (16 eq., corresponding to 2.0 eq. per cysteine; 32 µL, 10 mM in DMF) and PBS (65 µL, to achieve a final DMF concentration of 10%) were added. The reaction was carried at 25ºC, and its progress was monitored by reducing LC-MS. After completion of the reaction, excess **ET-CORM** was removed by ultrafiltration/diafiltration (UF/DF) using an Amicon® 3 kDa MWCO spin filter, with three buffer exchanges into PBS (pH 7.4). This was followed by two additional desalting steps into PBS (pH 7.4) using 7 kDa MWCO Zeba™ spin desalting columns, yielding the final stock solution in PBS (38.0 µM, 220 µL, 68% yield for **ET-CORM–Ab**).

5.2. Bioconjugate stability

PBS stability. The bioconjugate **ET-CORM–Ab** was diluted to a final concentration of 5 µM in PBS (pH 7.4) and incubated at 37 °C for the desired duration. Following incubation, the sample was reduced with DTT using standard protocols, and subsequently analyzed by LC-MS.

6. Cell experiments.

General conditions. Cells (SKBR3, MCF7) were acquired from ATCC and were grown in a humidified incubator at 37 ºC under 5% CO_2 with 90% humidity and split at approximately 80% confluence using Gibco™ TrypLE™ Express (cat. no 12604013) in order to keep them in the exponential growth phase. All cell cultures were grown in high glucose Gibco™ DMEM (+ pyruvate, GlutaMax™, cat. no 61965026) supplemented with 10% heat inactivated FBS (F9665, Sigma-Aldrich). Cells were counted using Countess™ 2 automated cell counter using Cell Counting Chamber Slides Invitrogen™ (C10315) and staining dead cells with trypan blue. Each experiment was repeated three times, in case of viability assays, each condition had three technical replicates.

6.1. Cell viability assays

Cells were seeded in Corning Costar 96-well clear flat-bottom plates at a density of 5,000 cells per well in 100 µL of media on day prior to the experiment. On the following day, test compounds were diluted to 4× the desired final concentration in 50 µL of media and added to the wells, followed by the addition of 50 µL of fresh media to reach the final volume. Cells were incubated for 48 hours (or as otherwise stated)

before the viability reagent was added (CellTiter-Blue® Cell Viability Assay G8080) and the fluorescence readout was measured after another 2 hours incubation at 37 ºC (λ_{ex} 540 nm, λ_{em} 590 nm) with Molecular Devices SpectraMax MiniMax 300 Imaging cytometer i3x. Cell viability % was calculated as 100% × F_{cells} / $F_{vehicle\_control}$, where F refers to the fluorescence of a given well. Vehicle-treated cells and cells treated with 100 µM digitonin were used as the negative (100% viability) and positive (0% viability) controls, respectively.

6.2. Cell imaging

General. Cells of interest were routinely passaged according to the recommendations and seeded one day before the experiment at a density of 120,000 cells/well, on 13 mm round cover slips (# 1.5, 0.16–0.19 mm) that were placed in 24-well plates and coated with poly-D lysine (Gibco™ cat. no A3890401, 30 min coating, washing with PBS (3×) and air drying).
For CO release studies in solution, the **1-Ac** probe was synthesized following the procedure described in the literature,[4] and a 10 mM stock solution (2000×) was prepared in DMSO. Before the experiment, the stock solution was further diluted 10-fold with DMSO (to 200×) and then filtered through a sterile 0.22 µm filter.
On the day of the experiment, cells at approximately 70% confluence were washed once with PBS (pH 7.4), then incubated with the **1-Ac** probe (5 µM, 0.5% DMSO) in fresh serum-free DMEM for 30 min. After incubation, cells were washed twice with PBS and treated with the desired concentration of CORM compound or a vehicle control. Small molecules were incubated for 60 minutes at µM concentrations, while bioconjugates such as **ET-CORM–Ab** were incubated for 2 hours at nM concentrations. A positive control (CORM3[7], 50 µM) was used to determine the maximum achievable turn-on fluorescence signal from the CO probe, allowing adjustment of laser intensity for imaging. Following treatment, cells were rinsed with PBS (3×) and fixed with 4% paraformaldehyde in PBS (300 µL / well) for 20 min, protected from light. Coverslips were then mounted onto glass slides using Mounting Medium with DAPI (abcam Fluoroshield cat. no AB104139). Images were acquired using the following channels: nucleus (λ_{ex} 405 nm, λ_{em} 460-490 nm) and CO-probe fluorescence (λ_{ex} 561 nm, λ_{em} 570-620 nm).
Confocal images were acquired on a Leica DMi8 microscope using a 40× oil-immersion objective, while conventional fluorescence images were obtained with an EVOS M5000 imaging system. For each sample, at least 3 images were taken at randomly assigned fields, at least three independent biological replicates were performed in each experiment unless stated otherwise. For visualization and analysis, raw images were imported using "default" color mode into Fiji ImageJ2 (version 2.14.0/1.54f). The fluorescence per cell were measured by creating binary image from CO-probe stain and selecting regions of interest (ROI) above 100 µm^2 and then using *measure* function.
For **1-Ac** mean fluorescence per cell quantification, the binary image was created from 561 nm channel. For comparative purposes and pictorial analysis, the maximum color for CO-channel was manually set to a value of 100 for every image using the color balance tool, while nuclei channel was adjusted automatically.
N.B. For experiments involving HER2 receptor blockade, cells were first incubated with trastuzumab (100 nM, 250 µL in complete media) for 30 minutes. After incubation, cells were washed three times with PBS to remove unbound antibody, and the experiment was then carried out as described above.

6.3. Flow cytometry: ET-CORM–Ab binding to the receptor using secondary antibody control

Cells were routinely cultured in complete DMEM medium, and split every other day when the cells reached 80% confluency in order to keep them in the exponential growth phase. On the day of the experiment, cells were harvested for staining of the HER2 receptor. Cells were resuspended at a concentration of 10^6 cells/mL in Flow cytometry buffer (Invitrogen™ eBioscience™ cat. no 00-4222-26)

to minimize non-specific binding and kept on ice for 1 hour. Cells were then incubated with either **ET-CORM–Ab** or **trastuzumab** control (100 nM) for 1 hour at 4 ºC. After three washes with PBS to remove unbound antibody, cells were stained with goat anti-human IgG–Alexa Fluor 647 (Invitrogen, A21445; 1:200) for 30 min at 4 °C, following the manufacturer's instructions. Non-stained controls and secondary antibody controls were also included. Cells were washed three times, resuspended in Flow cytometry buffer and filtered through cell strainer (40 μM) to remove cell clusters. Samples were analyzed by flow cytometer straight away counting at least 40,000 events per sample. The samples were acquired using CytoFLEX (Beckman Coulter) using routine methods. The data was analyzed by FlowJo software and only single cell data is shown. Washing refers to centrifugation of the sample at 200×*g*, 5 min, at 4ºC.

7. NMR spectra

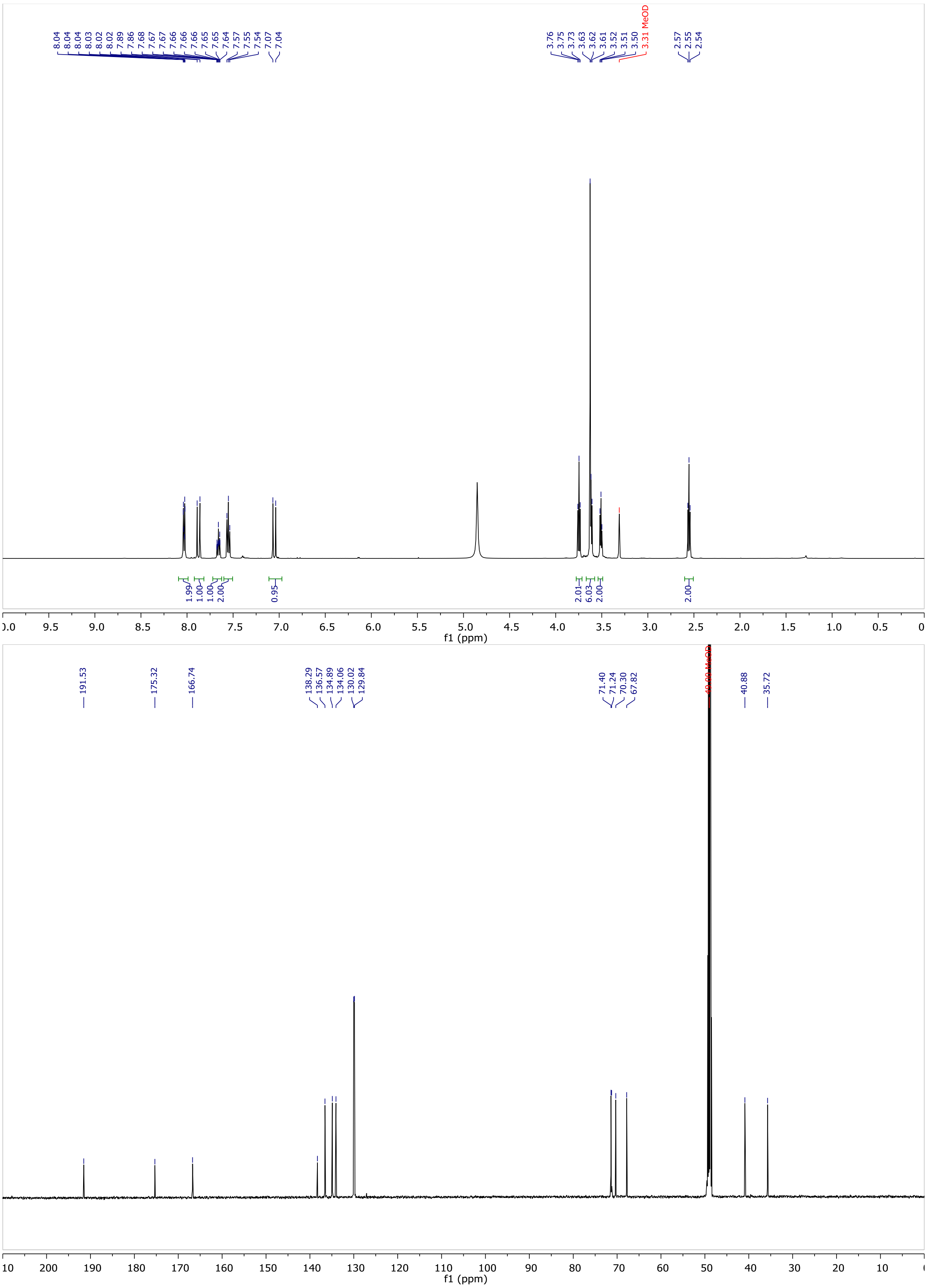

Figure S 11 ^{1}H and ^{13}C NMR spectra of compound **3** in CD_3OD.

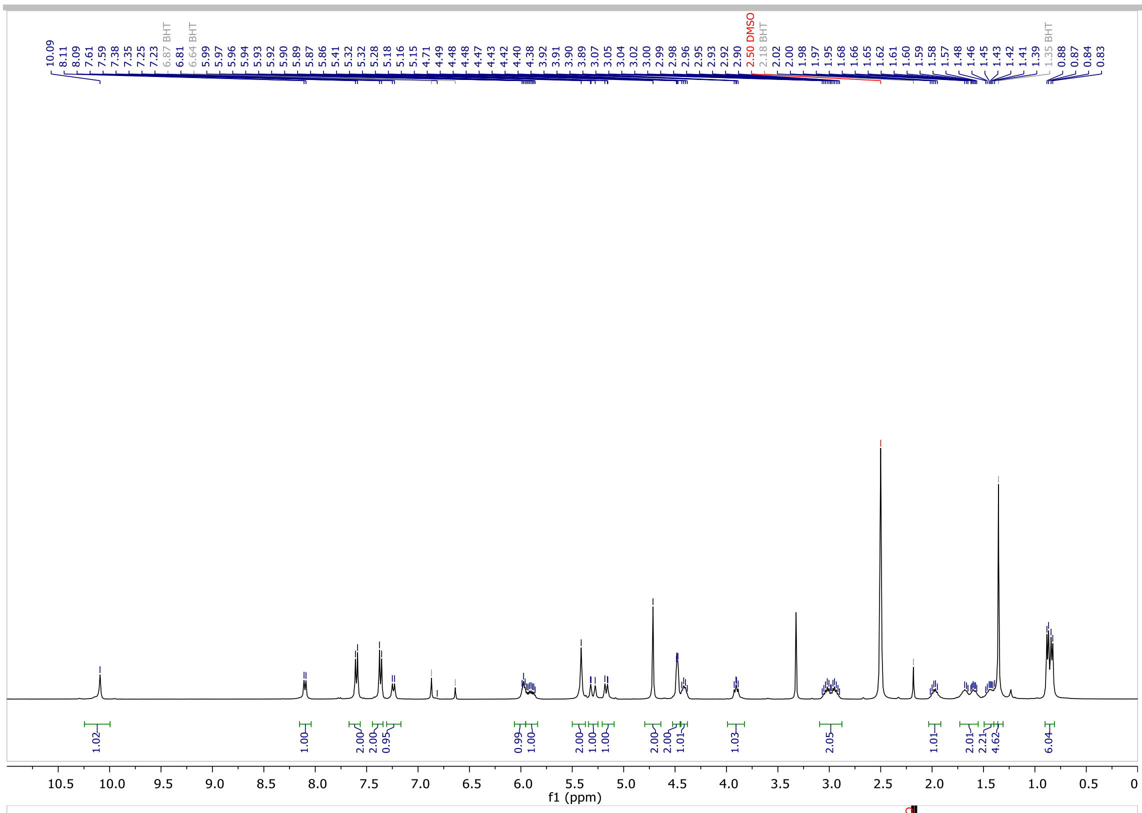

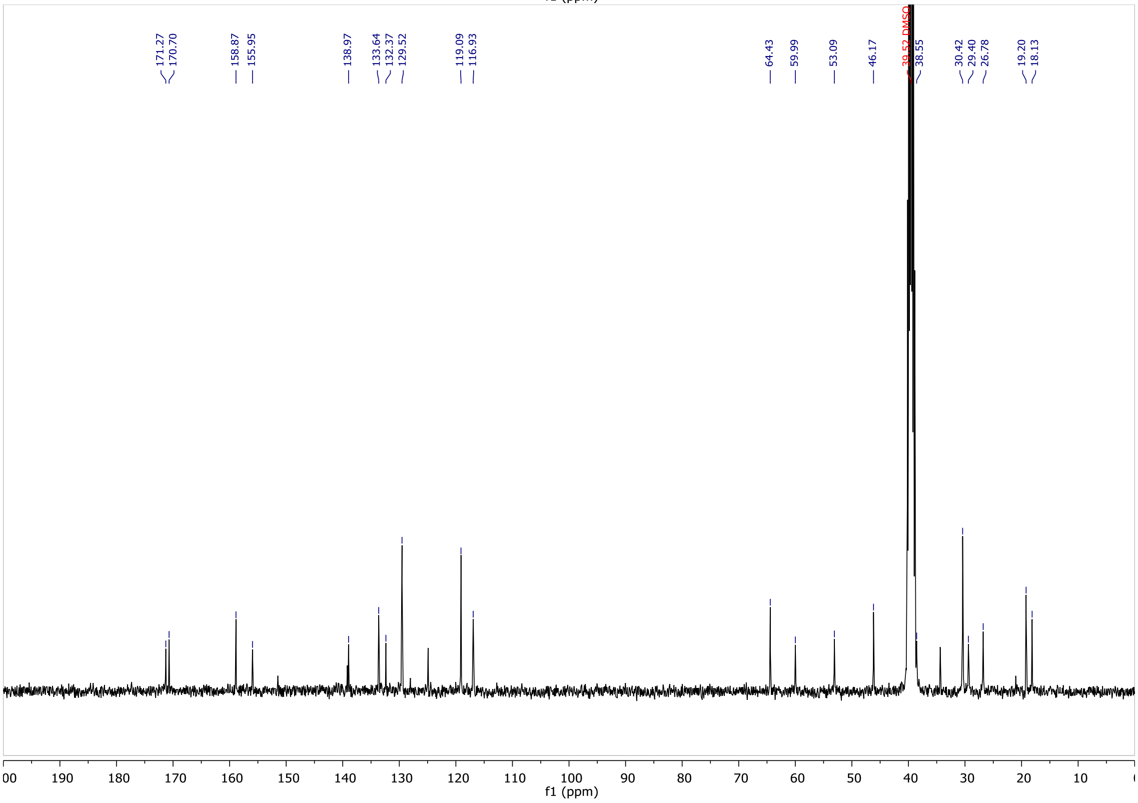

Figure S 12 ^{1}H and ^{13}C NMR spectra of compound **4** in DMSO-d_6

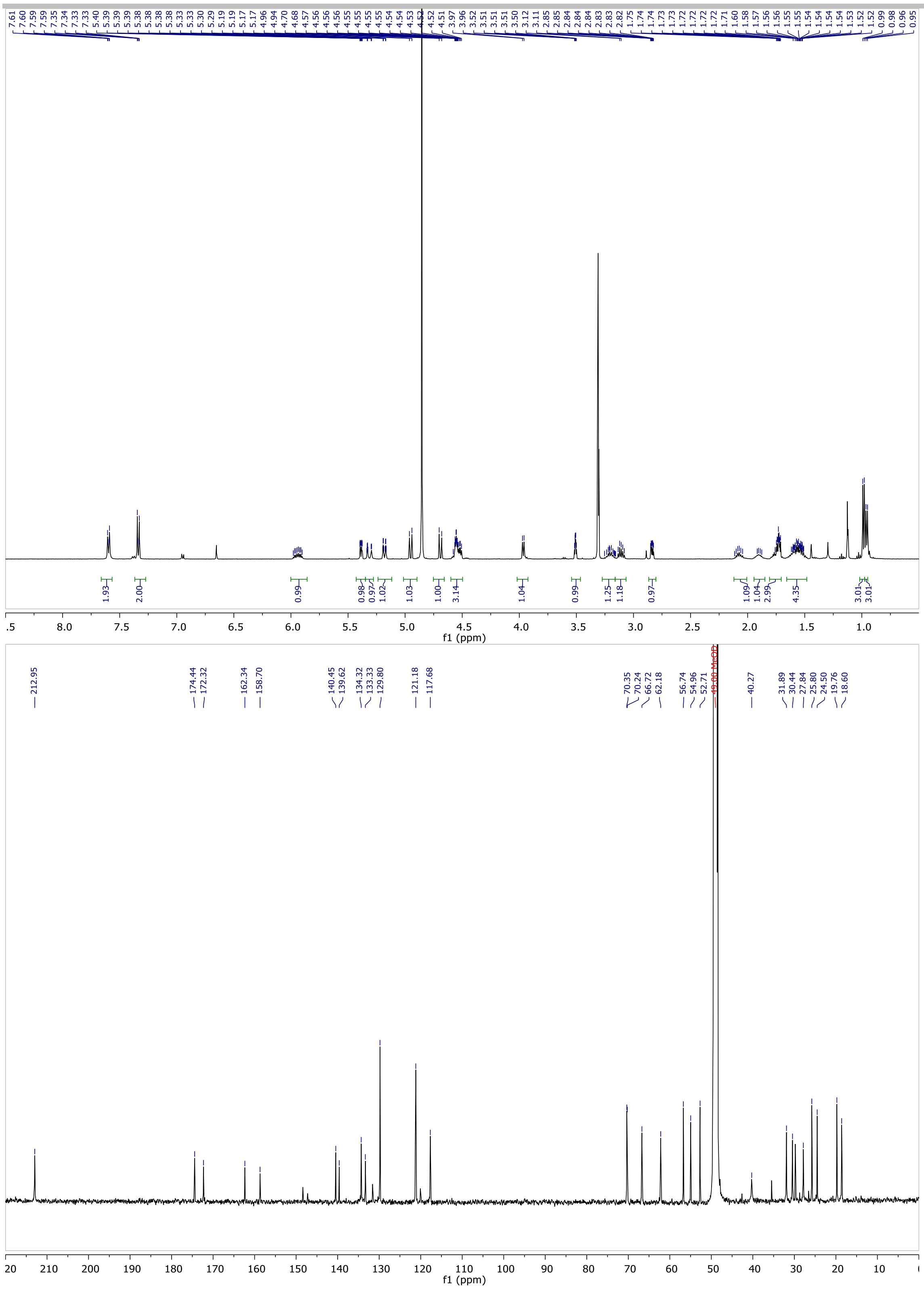

Figure S 13 ^{1}H and ^{13}C NMR spectra of compound **6** in CD_3OD.

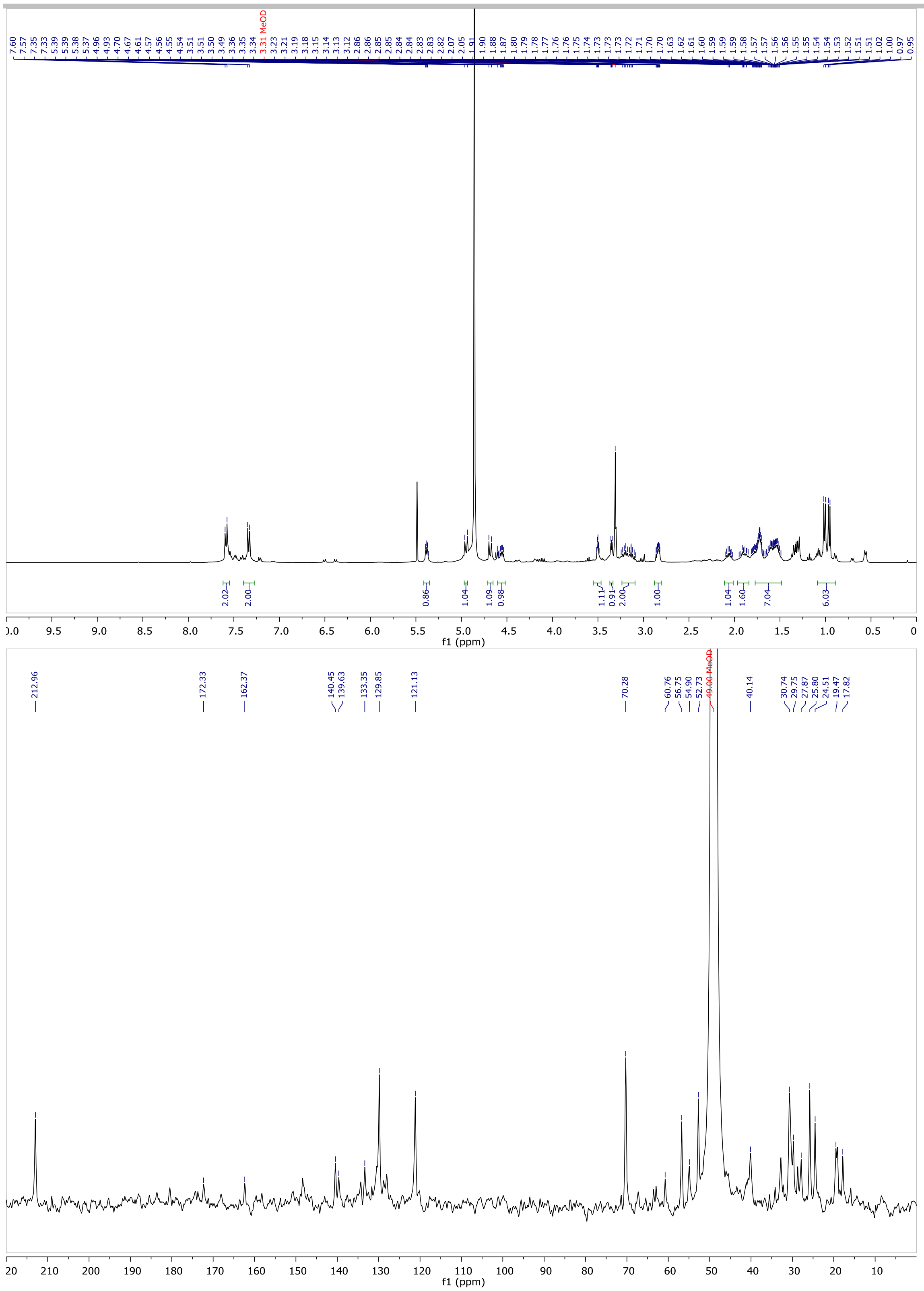

Figure S 14 ^{1}H and ^{13}C NMR spectra of compound **7** in CD_3OD.

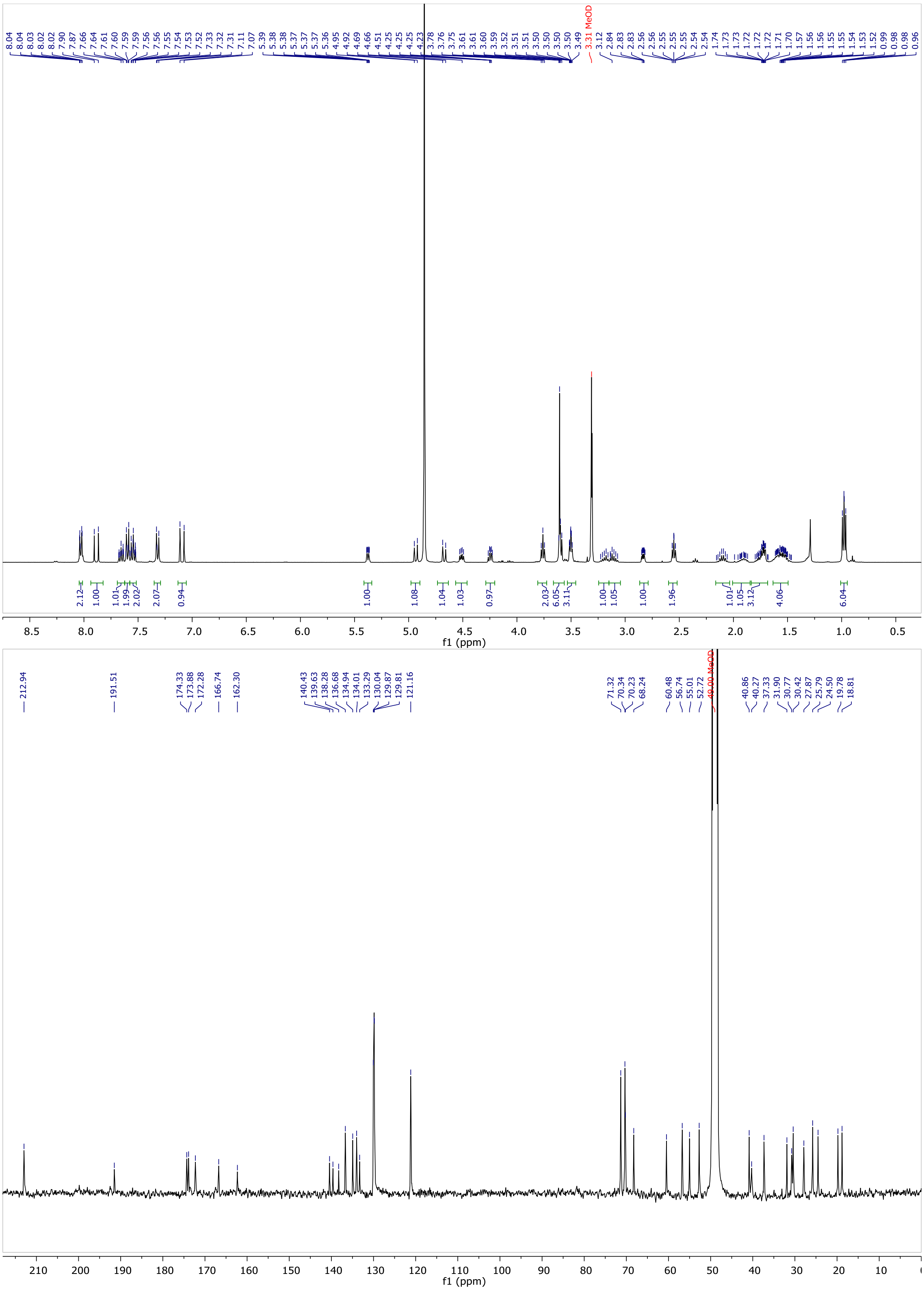

Figure S 15 ^{1}H and ^{13}C NMR spectra of compound **ET-CORM** in CD_3OD.

8. Protein LC-MS spectra

LC-MS of Unconjugated Trastuzumab

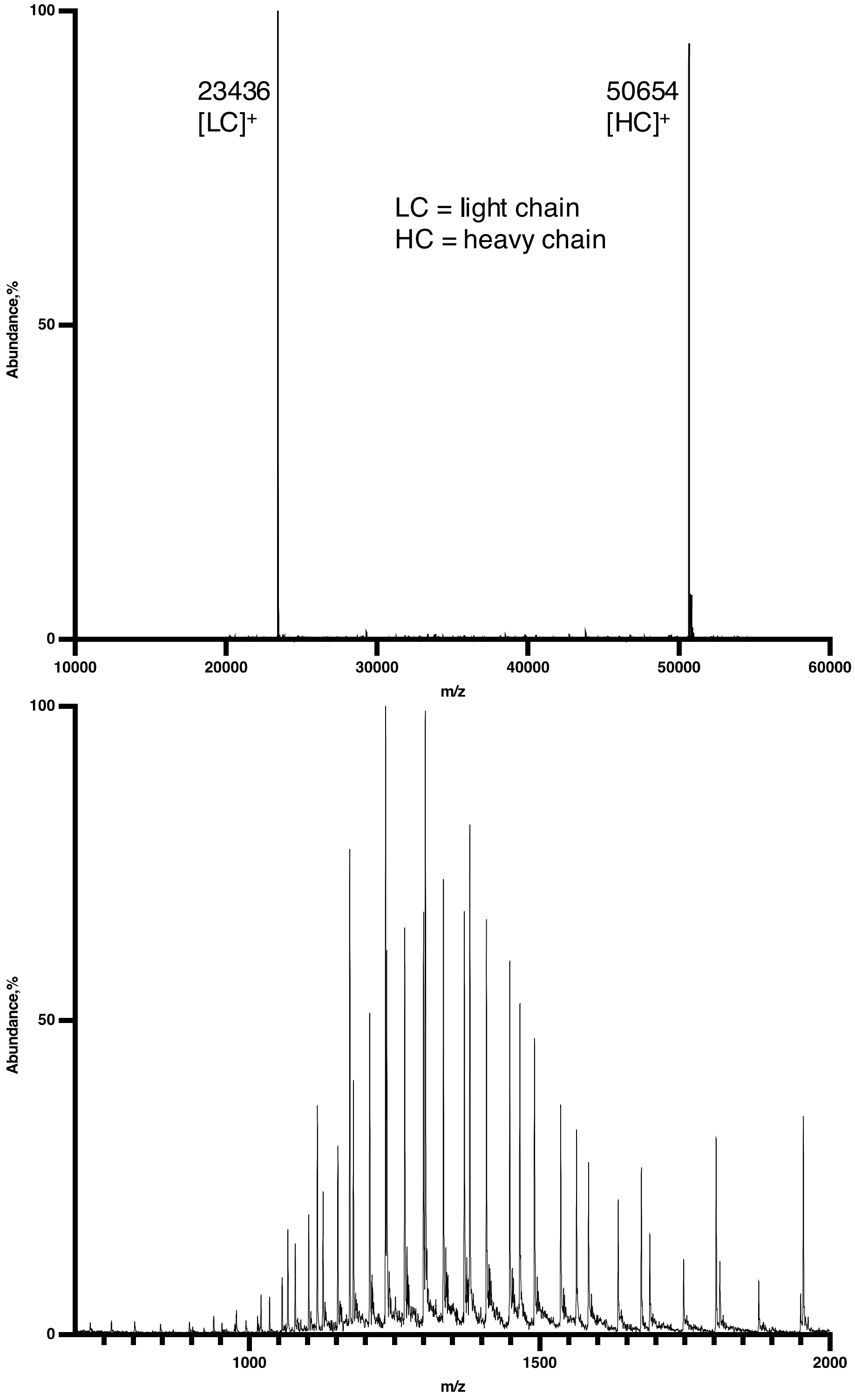

Figure S 16 LC-MS Data for pure commercially sourced reduced Trastuzumab antibody.

LC-MS of **ET-CORM–Ab**

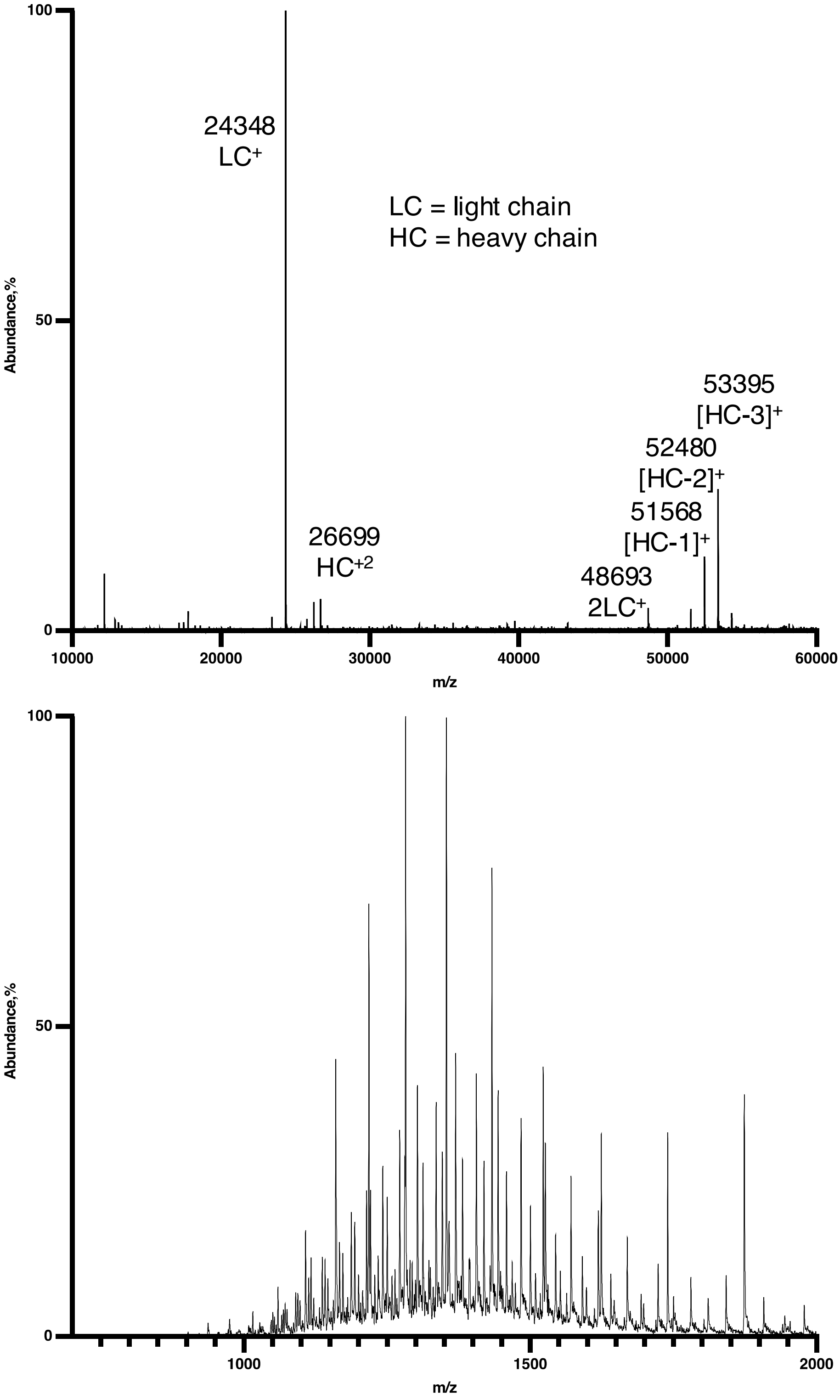

Figure S 17 LC-MS spectra of **ET-CORM–Ab**, ion series and deconvoluted spectra (calcd. For LC 24,349 Da, HC (3× modifications) 53,396 Da)

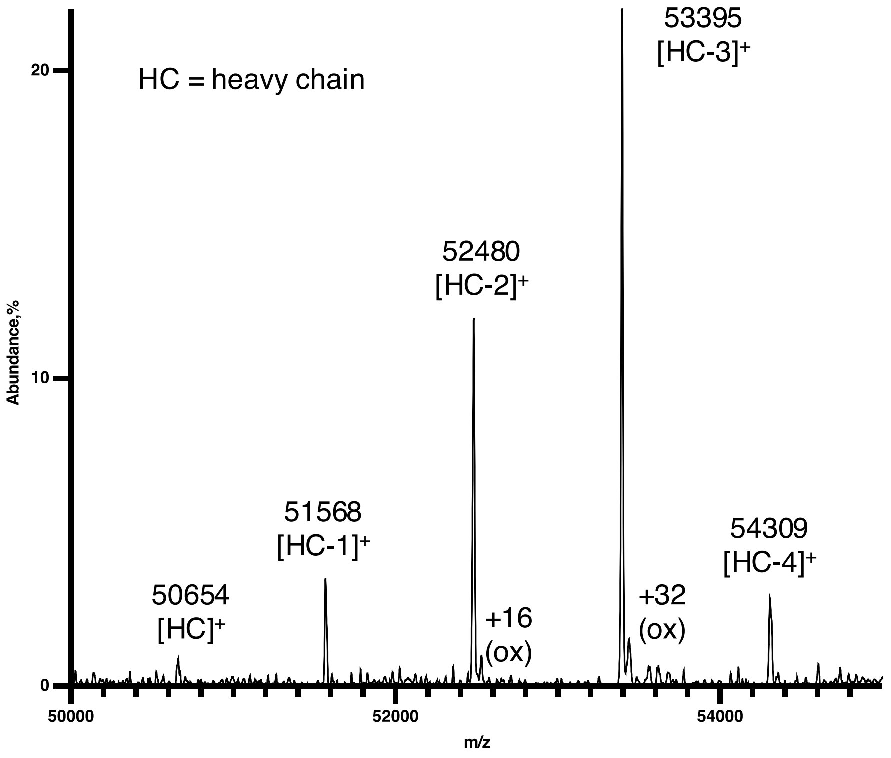

Figure S 18 Enlarged LC-MS spectrum of the **ET-CORM–Ab** heavy chain showing various modification levels (0-4), including an undesired fourth modification likely occurring at a lysine side chain.

9. Uncropped SDS-PAGE

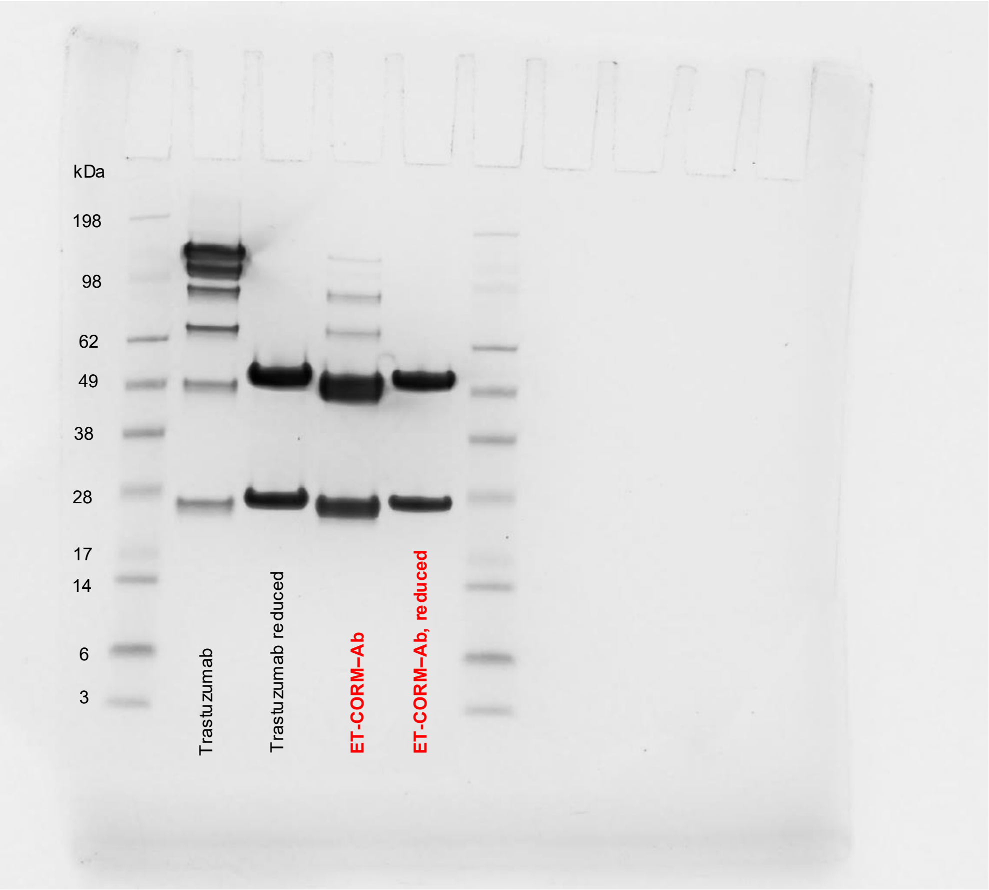

Figure S 19 Uncropped SDS-PAGE gel used in Figure 2

10. References

[1] N. S. Sitnikov, Y. Li, D. Zhang, B. Yard, H. G. Schmalz, *Angew. Chem. Int. Ed.* **2015**, *54*, 12314-12318.

[2] B. L. Oliveira, B. J. Stenton, V. B. Unnikrishnan, C. R. de Almeida, J. Conde, M. Negrão, F. S. S. Schneider, C. Cordeiro, M. G. Ferreira, G. F. Caramori, J. B. Domingos, R. Fior, G. J. L. Bernardes, *J. Am. Chem. Soc.* **2020**, *142*, 10869-10880.

[3] S. Romanski, B. Kraus, M. Guttentag, W. Schlundt, H. Rücker, A. Adler, J.-M. Neudörfl, R. Alberto, S. Amslinger, H.-G. Schmalz, *Dalton Trans.* **2012**, *41*, 13862-13875.

[4] K. Liu, X. Kong, Y. Ma, W. Lin, *Angew. Chem. Int. Ed.* **2017**, *56*, 13489-13492.

[5] A. Maeda, J. Bu, J. Chen, G. Zheng, R. S. DaCosta, *Mol. Imaging* **2014**, *14*, e7290.2014.00043.

[6] B. Bernardim, M. J. Matos, X. Ferhati, I. Compañón, A. Guerreiro, P. Akkapeddi, A. C. B. Burtoloso, G. Jiménez-Osés, F. Corzana, G. J. L. Bernardes, *Nat. Protoc.* **2019**, *14*, 86-99.

[7] J. E. Clark, P. Naughton, S. Shurey, C. J. Green, T. R. Johnson, B. E. Mann, R. Foresti, R. Motterlini, *Circ. Res.* **2003**, *93*, e2-8.
